# Single-cell quantification reveals divergent mixotrophic strategies underlying niche partitioning in marine *Synechococcus*

**DOI:** 10.1126/sciadv.aef8347

**Published:** 2026-08-21

**Authors:** Bowen He, Yanting Liu, Qi Chen, Tao Liu, Ta-Hui Lin, Silver Sung-Yun Hsiao, Der-Chuen Lee, Aixing Guo, Yuan Shen, Nianzhi Jiao, Qiang Zheng

**Affiliations:** ^1^ State Key Laboratory of Marine Environmental Science, College of Ocean and Earth Sciences, Xiamen University, Xiamen 361102, China.; ^2^ Fujian Key Laboratory of Marine Carbon Sequestration, Xiamen University, Xiang’an Campus, Xiang’an South Road, Xiamen 361102, China.; ^3^ Nansha Islands Coral Reef Ecosystem National Observation and Research Station, Guangzhou 510300, China.; ^4^ Key Laboratory of Functional and Clinical Translational Medicine, Xiamen Medical College, Universities of Fujian Province, Xiamen, Fujian, China.; ^5^ Institute of Astronomy and Astrophysics, Academia Sinica, Taipei 11529, Taiwan.; ^6^ Institute of Earth Sciences, Academia Sinica, Taipei 11529, Taiwan.

## Abstract

Marine *Synechococcus* is among the most widespread and productive autotrophs in the ocean, yet the quantitative role of mixotrophy in different lineages remains poorly constrained. Here, we compared organic nitrogen (urea and leucine) and carbon (glucose) utilization in nutrient-depleted versus nutrient-rich *Synechococcus* lineages by combining NanoSIMS-based single-cell measurements from field and laboratory incubations with omics analyses. Our findings revealed distinct mixotrophic strategies in different lineages. In nutrient-depleted lineages, elevated urea uptake supplied approximately 40–63% of the estimated total nitrogen demand. This pattern aligned with genomic evidence of enhanced urea transport, particularly the up-regulation of the high-affinity urea transporter *DUR3* in low-nitrogen environments. In contrast, nutrient-rich lineages exhibited greater glucose uptake, although the amended organic substrates contributed only 2 to 4% to the estimated cellular carbon demand. These lineage-specific mixotrophic strategies underpinned niche partitioning in marine *Synechococcus*, refining our understanding of their trophic differentiation and its implications for marine biogeochemical cycling.

## INTRODUCTION

Marine *Synechococcus* is one of the most abundant and widespread photosynthetic organisms in the marine environment, with global population estimates exceeding 10^26^ cells and contributing up to 17% of global primary production (*1*). Beyond fixing carbon, *Synechococcus* releases organic matter that sustains heterotrophic microbial communities, forming a critical nutritional base for the microbial food web and playing a crucial role in marine biogeochemical cycles (*2*, *3*).

For decades, the ecological success and biogeochemical impact of *Synechococcus* were attributed almost exclusively to its photosynthetic capacity. However, this paradigm has been fundamentally reshaped by emerging evidence of its mixotrophic capability. Genomic analyses have revealed a rich genetic repertoire for organic substrate acquisition, including transporters for amino acids (e.g., the *aapJQMP* operon), urea (e.g., the *urtABCDE* genes), cyanate (e.g., the *cynABD* genes), and sugars (e.g., *glcH*) (*4*–*6*). Physiological studies confirm the active assimilation of diverse organic compounds (*7*, *8*) and suggest that mixotrophy contributes to the ecological success of *Synechococcus*. For instance, urea uptake can supply up to 50% of the cellular nitrogen (N) requirement for nutrient-depleted *Synechococcus* in the North Pacific Subtropical Gyre, providing a major alternative to inorganic N sources (*9*, *10*). Furthermore, by directly assimilating dissolved organic carbon (DOC) (C), *Synechococcus* not only gains a competitive advantage in the dark layer but also redefines its biogeochemical role, acting as both a producer and a consumer of fixed C (*11*).

The mixotrophy of *Synechococcus* has broadened its ecological significance for marine biogeochemical cycling, yet current understanding relies heavily on laboratory model strains (*5*, *12*, *13*) or geographically limited field studies (*8*, *9*, *14*). Critically, these approaches often overlook the extensive phylogenetic and physiological diversity within *Synechococcus*, which comprises multiple phylogenetic clades belonging to functionally distinct lineages (*4*). These lineages have evolved specific adaptations to thrive in contrasting nutrient niches, from nutrient-rich coastal waters (e.g., clades I, IV, V, CB4, and CB5) to nutrient-depleted open oceans (e.g., clades II and III) (*15*, *16*). This ecological divergence implies that their capacity for, and reliance on, mixotrophy are likely highly variable, with different implications in marine biogeochemical cycles.

To quantify mixotrophic potential across *Synechococcus* lineages, microbial communities from both nutrient-depleted (South China Sea) and nutrient-rich (Xiamen coastal waters) environments, as well as representative strains, were amended with isotope-labeled substrates and analyzed by nano-scale secondary ion mass spectrometry (NanoSIMS) following cell sorting. By integrating in situ observations with laboratory experiments, we assessed the stability of lineage-specific mixotrophic phenotypes across disparate nutrient regimes. In parallel, our multiomics analysis probed the genomic basis and transcriptional regulation of key urea transporters, notably the high-affinity transporter *DUR3*, in *Synechococcus* from low-N oceanic regions, providing a molecular explanation for these lineage-specific adaptations. Collectively, our results refine the lineage-resolved understanding of *Synechococcus* mixotrophy and provide quantitative evidence for how mixotrophic traits may contribute to niche differentiation and oceanic biogeochemical cycling.

## RESULTS

### Single-cell organic substrate uptake rates and net assimilation percentages of natural and cultured *Synechococcus*

**Fig. 1.**
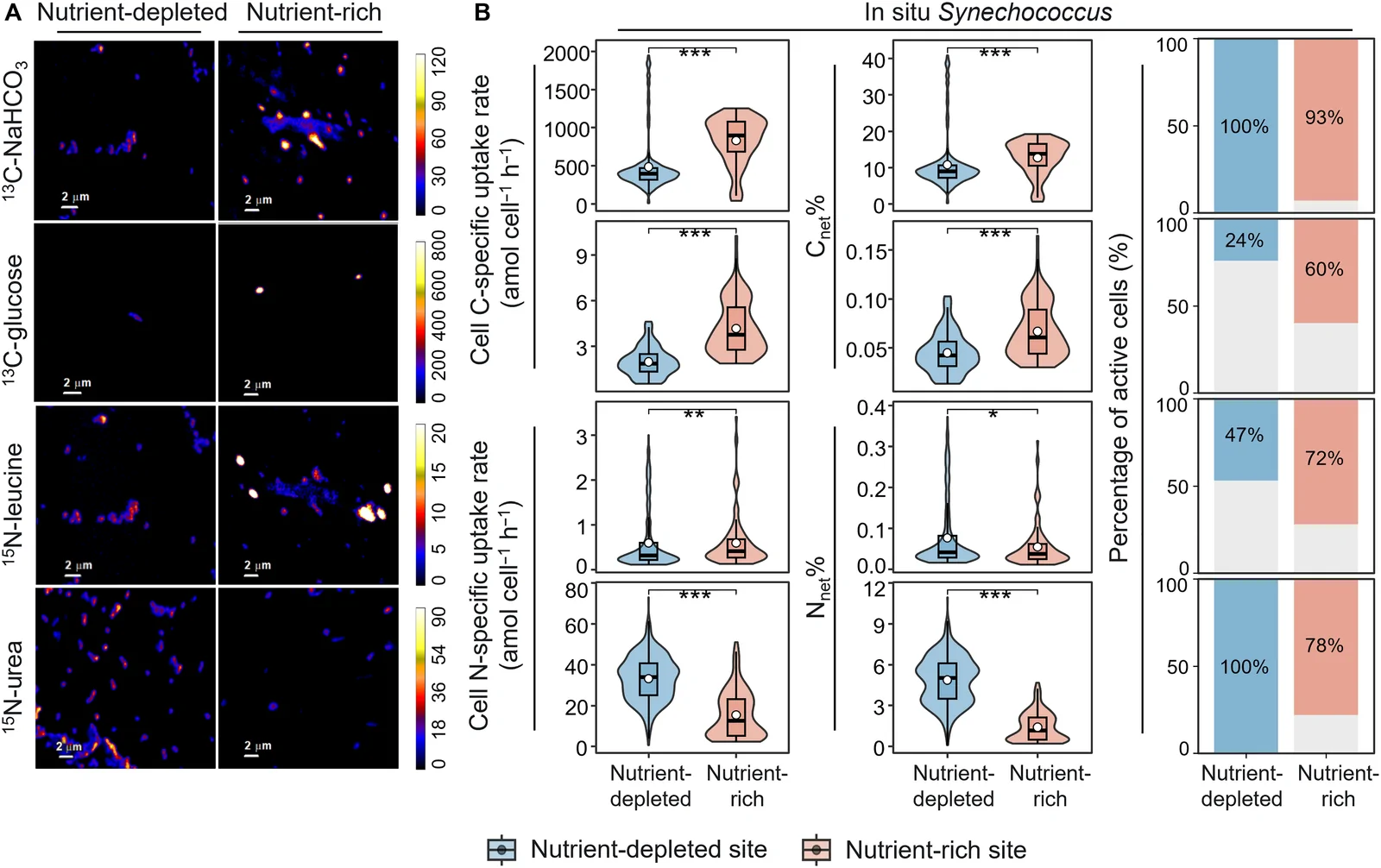
Examples of NanoSIMS images and single-cell C- and N-specific substrate assimilation by in situ *Synechococcus*. (**A**) Examples of NanoSIMS images showing ^12^C^13^C^−^ or ^12^C^15^N^−^ enriched *Synechococcus* cells from in situ nutrient-depleted and nutrient-rich sites. (**B**) Single-cell uptake rates (amol cell^−1^ hour^−1^), alongside cell-specific net C or N assimilation percentages (C_net_% or N_net_%) and the proportion of active cells for C-fixation (*n* = nutrient-depleted site: 1018; nutrient-rich site: 171), glucose (*n* = 141; 100), leucine (*n* = 476; 132), and urea (*n* = 591; 129) in *Synechococcus* from nutrient-depleted and nutrient-rich sites. Asterisks denote statistical significance (*P < 0.05, **P < 0.01, and ***P < 0.001).

To compare mixotrophic activity between nutrient-depleted and nutrient-rich *Synechococcus* populations, we quantified the proportion of active cells (reflecting uptake activity), cell-specific uptake rates (reflecting metabolic influx), and net assimilation percentages (C_net_% and N_net_%, reflecting the fraction of the total cellular C or N pool derived from newly assimilated substrate during the incubation period) in the nutrient-depleted South China Sea and nutrient-rich Xiamen coastal waters (Fig. 1 and tables S7 and S8). These sites featured contrasting nutrient conditions, with inorganic N (NO_3_^−^, NO_2_^−^, and NH_4_^+^) concentrations ∼100-fold lower and organic N (urea and leucine) ∼10-fold lower at the nutrient-depleted site (table S1).

Correspondingly, natural *Synechococcus* populations exhibited clear ecological differentiation in substrate assimilation. The nutrient-depleted *Synechococcus* more actively assimilated urea (100% active versus 78% active) at markedly higher rates than nutrient-rich populations [median: 33.84 (Q1: 25.14, Q3: 40.72) versus 12.56 (5.29, 23.12) amol N cell^−1^ hour^−1^, *P* = 1.73 × 10^−38^]. This enhanced uptake rate resulted in a much higher N_net_% from urea in the nutrient-depleted populations compared to the nutrient-rich ones [5.03% (3.49, 6.1) versus 1.15% (0.48, 2.12), *P* = 1.11 × 10^−59^]. Leucine assimilation remained consistently low in both environments [nutrient-depleted: 0.31 (0.22, 0.6) amol N cell^−1^ hour^−1^; nutrient-rich: 0.41 (0.27, 0.67) amol N cell^−1^ hour^−1^, *P* = 0.0052], accounting for a median of only 0.04% of the total cellular N pool at both sites. Conversely, nutrient-rich populations exhibited more active (60% versus 24%) and higher glucose uptake than nutrient-depleted *Synechococcus* [2.73 (1.92, 3.65) versus1.35 (0.94, 1.96) amol C cell^−1^ hour^−1^, *P* = 1.21 × 10^−24^]. This led to a higher but overall marginal glucose-derived C_net_% in the nutrient-rich water [0.06% (0.05, 0.09) versus 0.02% (0.02, 0.03), *P* = 6.56 × 10^−10^].

**Fig. 2.**
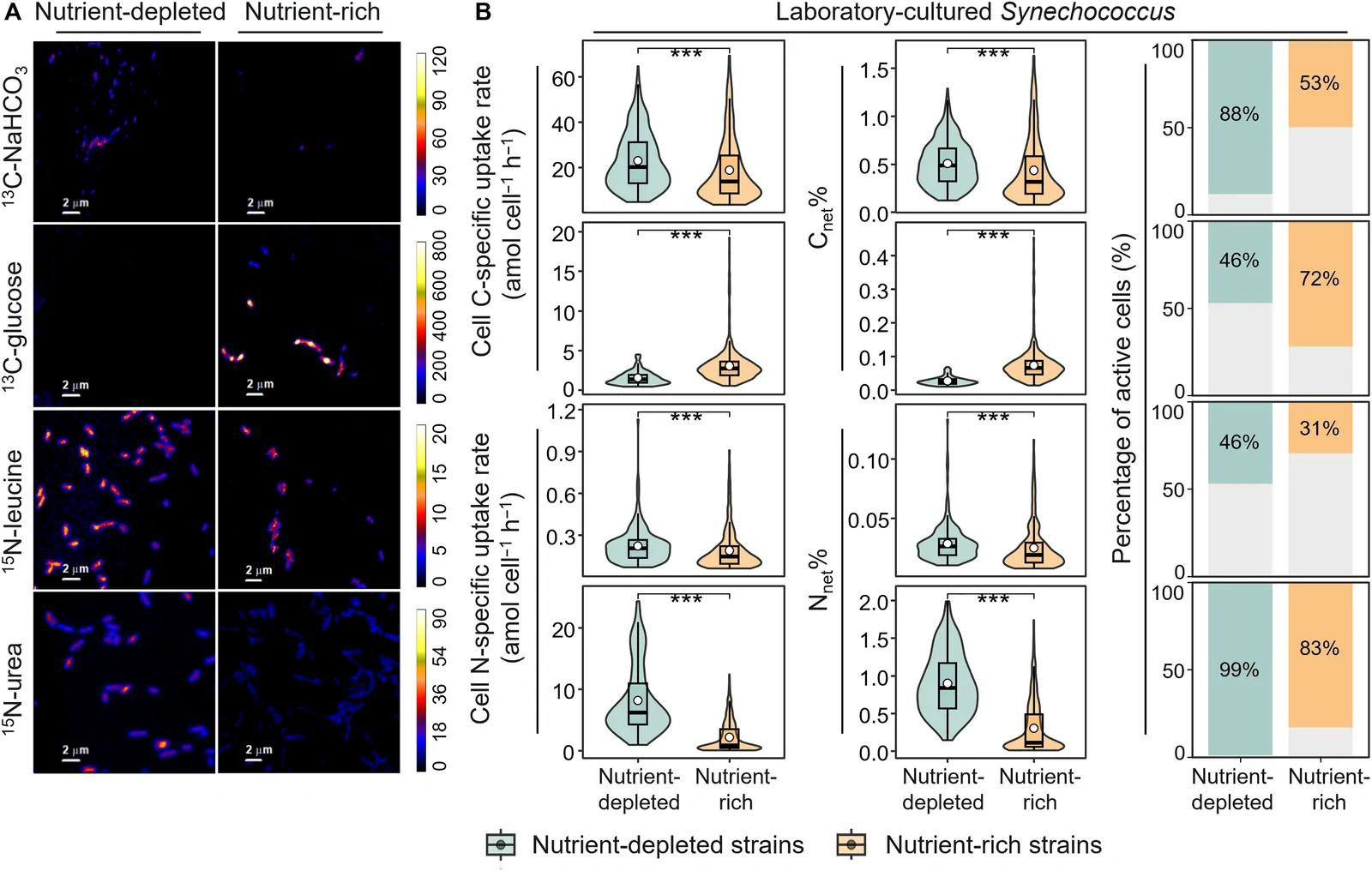
Examples of NanoSIMS images and single-cell C- and N-specific substrate assimilation by laboratory-cultured *Synechococcus*. (**A**) Examples of NanoSIMS images showing ^12^C^13^C^−^ or ^12^C^15^N^−^ enriched cells of laboratory-cultured nutrient-depleted (*Synechococcus* sp. YX04-3) and nutrient-rich (*Synechococcus* sp. CC9311) strains. (**B**) Single-cell uptake rates (amol cell^−1^ hour^−1^), alongside cell-specific net C or N assimilation percentages (C_net_% or N_net_%) and the proportion of active cells (%) for C-fixation (*n* = nutrient-depleted strains: 489; nutrient-rich strains: 436), glucose (*n* = 134; 654), leucine (*n* = 259; 255), and urea (*n* = 291; 749) in nutrient-depleted and nutrient-rich *Synechococcus* strains. Asterisks denote statistical significance (***P < 0.001).

Laboratory-cultured *Synechococcus* strains displayed patterns consistent with field observations (Fig. 2 and tables S7 and S8). Under comparable culture conditions, nutrient-depleted strains still showed more active (99% versus 83%) and significantly higher urea uptake than nutrient-rich strains [6.19 (4.29, 10.96) versus 0.81 (0.42, 3.49) amol N cell^−1^ hour^−1^, *P* = 7.07 × 10^−81^], leading to a higher urea-derived N_net_% [0.84% (0.57, 1.17) versus 0.11% (0.06, 0.49), *P* = 9.04 × 10^−79^]. In contrast, nutrient-rich strains exhibited more active (72% versus 46%), greater glucose uptake [2.73 (1.92, 3.65) amol cell^−1^ hour^−1^ versus 1.35 (0.94, 1.96) amol cell^−1^ hour^−1^, *P* = 3.88 × 10^−29^] and higher glucose-derived C_net_% [0.06% (0.05, 0.09) versus 0.02% (0.02, 0.03), *P* = 3.66 × 10^−50^].

To place *Synechococcus* mixotrophy in the context of coexisting heterotrophic bacteria, we compared substrate uptake rates, C_net_%, and N_net_% between *Synechococcus* and heterotrophic bacteria (figs. S4 and S5, and tables S9 and S10). Across paired experiments, *Synechococcus* exhibited substantially higher cell-specific uptake rate and N_net_% from urea than heterotrophic bacteria, particularly in nutrient-depleted waters [38.35 (34.02, 44.45) versus 3.57 (0.30, 8.38) amol cell^−1^ hour^−1^; 5.75% (5.10, 6.66) versus 1.71% (0.14, 3.9)]. In contrast, heterotrophic bacteria exhibited higher glucose uptake and glucose-derived C_net_% than *Synechococcus*, particularly in nutrient-rich waters [4.79 (1.90, 13.43) versus 3.76 (2.76, 5.56) amol cell^−1^ hour^−1^; 0.39% (0.15, 1.09) versus 0.06% (0.04, 0.09)].

### Mixotrophic N and C transporter expression in *Synechococcus* between the nutrient-depleted and nutrient-rich study sites

**Fig. 3.**
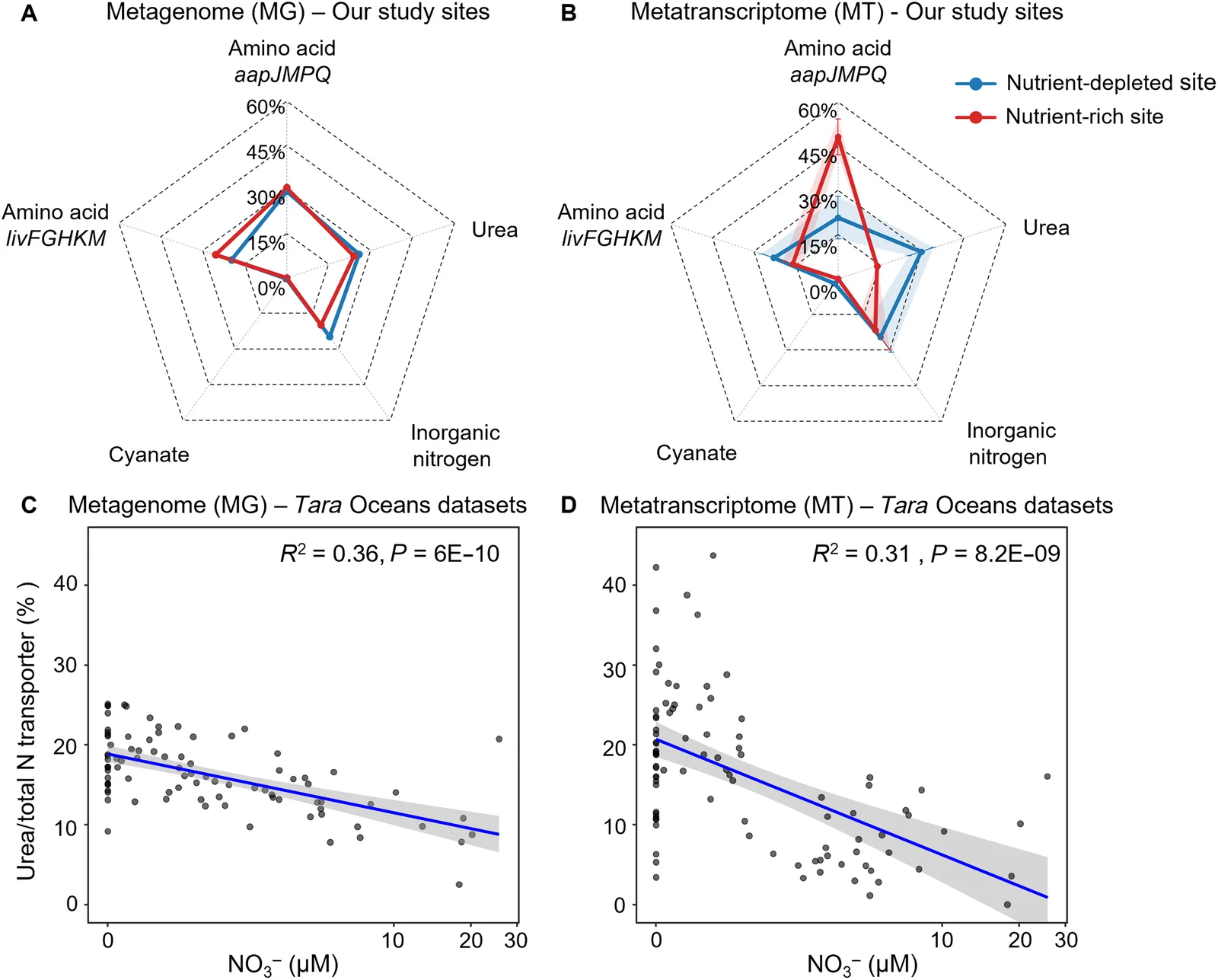
Relative importance of urea transporters (*urtABCDE* and *DUR3*) among different N substrate transporters in *Synechococcus* from our study sites and *Tara* Oceans datasets. Radar plots quantify the (**A**) relative abundance and (**B**) expression of each N substrate transporter in *Synechococcus*, expressed as a percentage of the total N-substrate transporters (%). Blue and red represent the nutrient-depleted (*n* = 6, metatranscriptomic data) and nutrient-rich (*n* = 3, metatranscriptomic data) study site, respectively. Correlations between ambient NO_3_^−^ concentration and the contribution of urea transporters to total (**C**) relative abundance (*n* = 89) and (**D**) expression (*n* = 93) of different N substrate transporters in *Synechococcus* from the *Tara* Oceans prokaryotic database. Linear regressions of urea contribution versus NO_3_^−^ concentration are shown with 95% confidence interval (gray shadow).

The genetic basis of the distinct mixotrophic strategies was investigated by the relative abundance and expression of nutrient transporters in natural *Synechococcus* populations from the nutrient-depleted and nutrient-rich sites (Fig. 3, A and B). Transporters associated with uptake of inorganic N (nitrate, nitrite, and ammonium), organic N substrates (amino acids, cyanate, and urea), and glucose were analyzed (gene list in table S6).

**Fig. 4.**
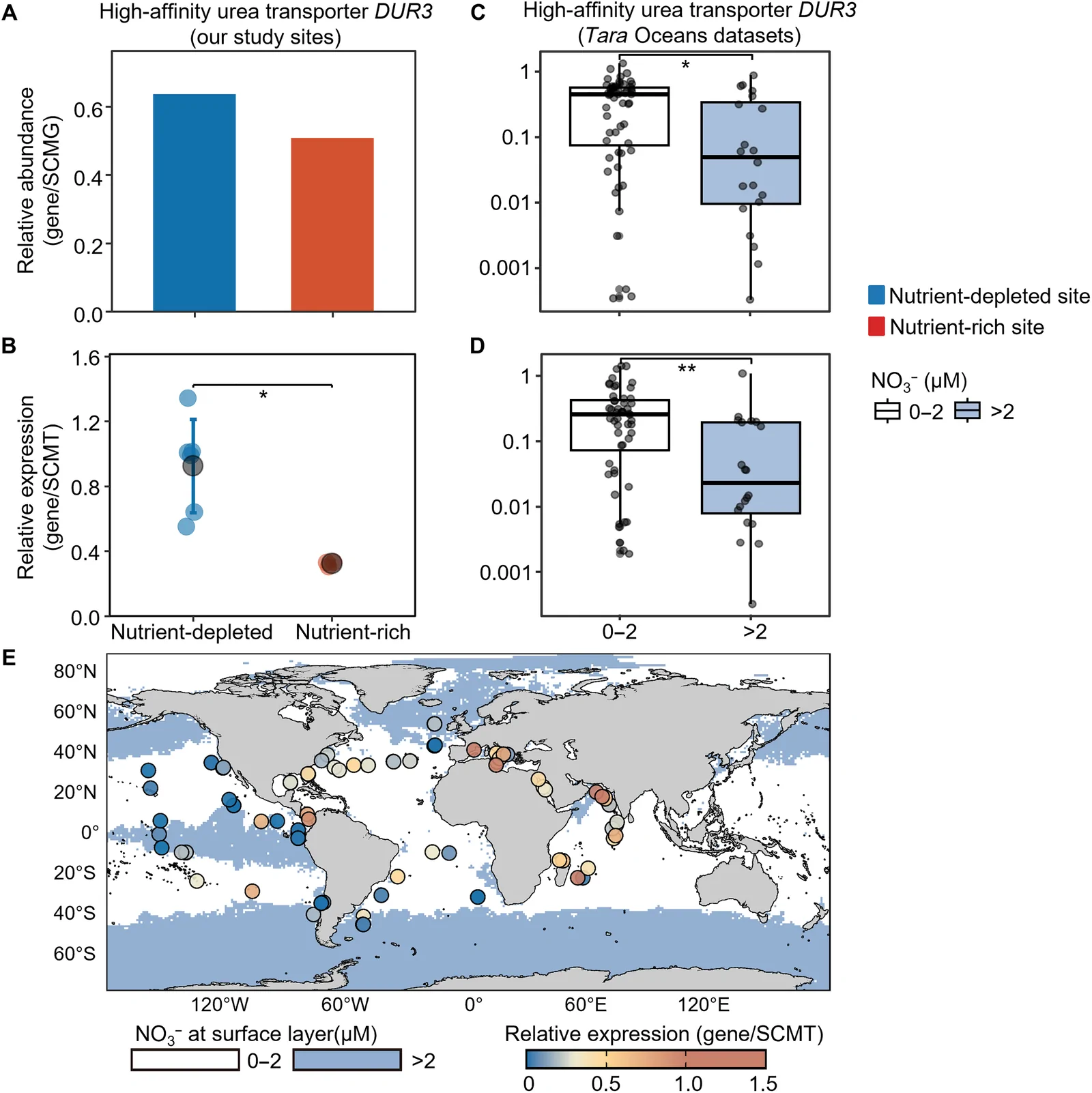
Relative abundance and expression of the high-affinity urea transporter *DUR3* in *Synechococcus* from our study sites and *Tara* Oceans datasets. (**A** and **B**) Relative abundance and expression of the urea transporter *DUR3* between nutrient-depleted (blue, *n* = 6, metatranscriptomic data) and nutrient-rich (red, *n* = 3, metatranscriptomic data) sites. Black dots denote mean values of relative expression, with error bars showing SD. (**C** and **D**) Boxplots of relative abundance (*n* = low-N: 56; high-N: 20) and expression (*n* = 52; 20) in low-N (NO_3_^−^ = 0 to 2 μM) and high-N (NO_3_^−^ > 2 μM) *Tara* Oceans regions. (**E**) Global NO_3_^−^ distribution and relative expression of the urea transporter *DUR3* across *Tara* Oceans stations (*n* = 72). Asterisks denote statistical significance (*P < 0.05 and **P < 0.01).

Metagenomic data showed similar relative abundances of N-substrate transport genes at both sites (Fig. 3A). However, urea transporters dominated the N-transport transcriptome at the nutrient-depleted site (Fig. 3B), where the urease operon also showed higher expression (*P* = 0.05; fig. S6). Notably, the high-affinity urea transporter *DUR3* (*17*) displayed higher abundance and expression (*P* = 0.02) at the nutrient-depleted site (Fig. 4, A and B). Amino acid transporters also exhibited site-specific patterns: The *livFGHKM* system accounted for a larger proportion of N-transport transcriptome at the nutrient-depleted site, whereas the *aapJMPQ* system was more highly represented at the nutrient-rich site (Fig. 3B). In addition, the cyanate transporter (*cynABD*), associated with the uptake of another important organic N source, showed much higher abundance and was expressed exclusively at the nutrient-depleted site (fig. S8, A and B). Within the nutrient-depleted populations, clade III (exclusive to this site; fig. S1) exhibited the highest relative abundance and expression levels for both urea and cyanate transport genes among all clades examined (fig. S9). The total glucose transporter expression in *Synechococcus* did not differ significantly between nutrient-depleted and nutrient-rich sites (fig. S10, A and B). However, the glucose permease *glcP*, one of the major glucose transporters, was more abundant and significantly highly expressed (*P* = 0.02) in nutrient-rich *Synechococcus* (fig. S10, E and F), consistent with the higher glucose uptake rates observed in situ.

### Global patterns of *Synechococcus* organic matter transport in *Tara* Oceans datasets

We further examined the *Synechococcus* substrate utilization patterns observed in cultures and regional field studies at a global scale using metagenomic and metatranscriptomic data from the *Tara* Oceans expedition. Specifically, we quantified the contribution of urea transporters to total N transport and assessed the expression of high-affinity urea, cyanate, and glucose transport genes (tables S11 and S12).

Across the global ocean, both metagenomic and metatranscriptomic data displayed a significant negative correlation between urea transporter contribution and ambient NO_3_^−^ concentration [metagenome: coefficient of determination (*R*^2^) = 0.36, *P* = 6 × 10^−10^; metatranscriptome: *R*^2^ = 0.31, *P* = 8.2 × 10^−09^; Fig. 3, C and D]. This pattern corresponded to higher abundance and expression of the high-affinity urea transporter *DUR3* (Fig. 4, C and D) and cyanate transporter (fig. S8, C and D) in low-N regions (NO_3_^−^ = 0 to 2 μM). The urease gene operon (with the exception of *ureC*) and amino acid transporters, however, exhibited nonsignificant changes across different regions (*P* > 0.05; figs. S6 and S7). While glucose transporters only showed a modest up-regulation in global high-N regions (NO_3_^−^ > 2 μM; *P* = 0.002) (fig. S10, C and D), the glucose permease *glcP* exhibited higher abundance and expression (*P* = 0.0002 and 0.05) in high-N regions (fig. S10, G and H).

### Organic substrate contribution to estimated *Synechococcus* cellular total C and N uptake

**Fig. 5.**
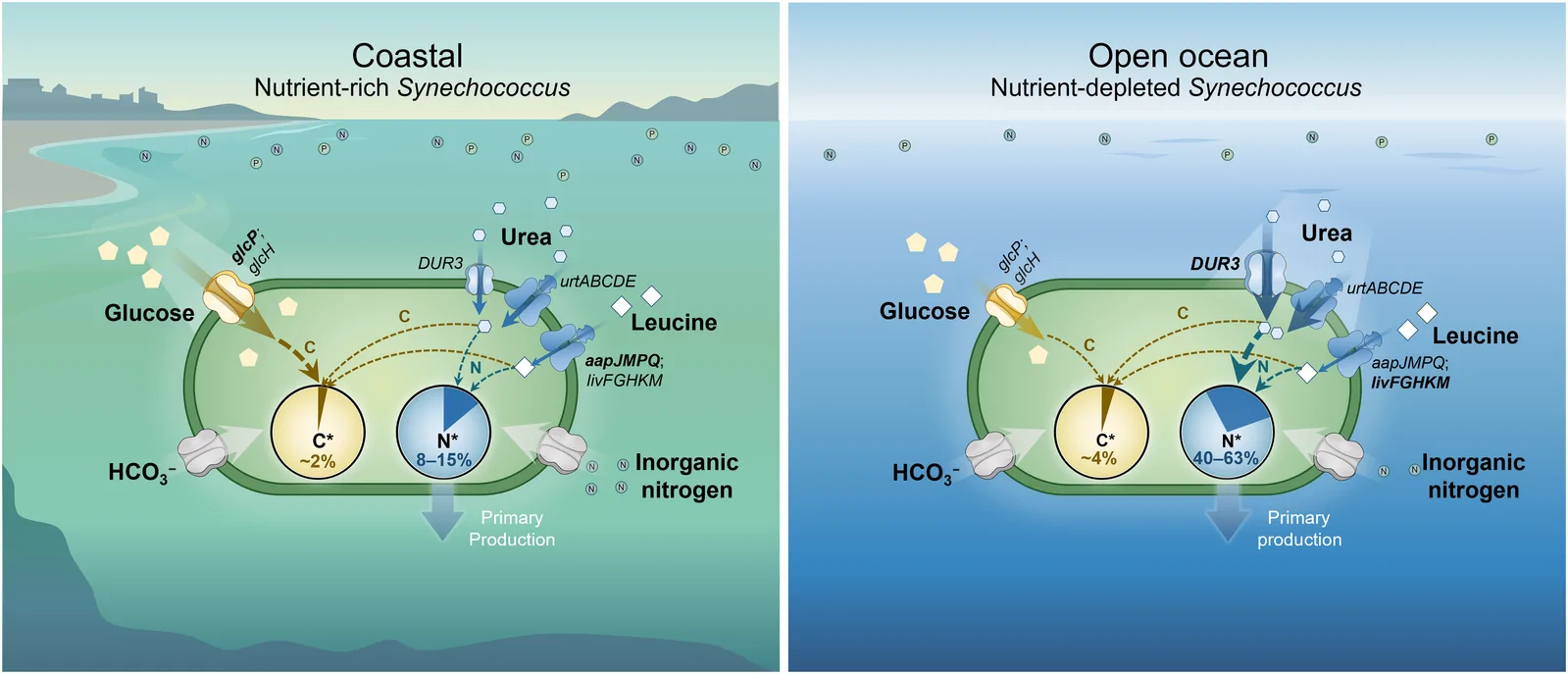
Schematic of C and N acquisition and contribution in *Synechococcus* from nutrient-rich (left) and nutrient-depleted (right) sites. C* and N* denote the estimated total cellular C and N uptake, respectively. In the schematic cells, the dark sectors of the pie charts indicate the proportion of organic C or N derived from amended substrates relative to the estimated total uptake. *Synechococcus* from nutrient-rich waters exhibited higher glucose uptake, whereas those in nutrient-depleted waters were more efficient at urea transport, a phenotypic trait consistent with the up-regulation of urea transporters, including high-affinity urea transporter *DUR3*. This enhanced urea uptake promotes the contribution of amended organic substrates to estimated cellular N demand (40 to 63%; indicated by N* pie charts) and to primary production (42 to 66%; bottom arrows) in nutrient-depleted *Synechococcus*. Conversely, the amended organic substrates contributed minimally (2 to 4%; C* pie charts) to the cellular carbon demand in both lineages.

Natural *Synechococcus* populations displayed distinct C and N acquisition patterns between sites (Fig. 5). Organic C derived from amended substrates (glucose, leucine, and urea) contributed minimally to estimated total C uptake, averaging ∼4% in nutrient-depleted populations and ∼2% in nutrient-rich populations. In contrast, organic N from amended substrates (leucine and urea), overwhelmingly attributable to urea, represented a substantial nutritional supplement, particularly for nutrient-depleted populations (40 to 63% of estimated total N demand), approximately fourfold higher than in nutrient-rich populations (8 to 15%).

## DISCUSSION

### Divergent mixotrophic strategies in nutrient-depleted and nutrient-rich *Synechococcus*

A fundamental distinction between nutrient-rich and nutrient-depleted *Synechococcus* lineages lies in their metabolic demands (*4*, *18*), which likely drives lineage-specific organic matter utilization patterns. Nutrient-rich lineages face fluctuating conditions driven by mixing and upwelling (*19*) and therefore prioritize energy acquisition to support sensing and acclimating to stress (*20*, *21*). These processes are energetically expensive, involving (ATP)–dependent two-component signaling systems, such as histidine kinase-response regulator pathways, and other molecular machinery necessary for environmental acclimation. Conversely, nutrient-depleted lineages experience severe nutrient limitation, particularly N, making nutrient acquisition their primary physiological challenge (*22*). This metabolic divergence aligns with previous studies showing that energy-limited cells near the base of the photic zone rely heavily on organic C (*23*), while N-limited cells in gyres draw substantially on organic N (*9*). Collectively, these results suggest a general mechanism whereby lineages preferentially exploit organic substrates to relieve their primary physiological limitation.

Our field observations are consistent with these ecological pressures. The nutrient-depleted South China Sea sites had approximately 100-fold lower inorganic N and a markedly higher proportion of organic N (∼22% versus ∼2%) than the nutrient-rich Xiamen site (table S1), creating selective conditions that favor adaptive evolution toward organic N utilization. Correspondingly, *Synechococcus* from the nutrient-depleted site showed markedly enhanced urea uptake and greater incorporation of urea-derived N into biomass than nutrient-rich populations. NanoSIMS analyses revealed that nutrient-depleted *Synechococcus* showed significantly higher urea uptake activity (100% versus 78%), rates (*P* = 1.73 × 10^−38^), and urea-derived N_net_% (*P* = 1.11 × 10^−59^) than nutrient-rich populations, while leucine uptake remained uniformly low at both sites (Fig. 1 and tables S7 and S8).

*Synechococcus* is known to synthesize and release N-rich organic metabolites (*24*), indicating a sustained N demand that likely selects for substrates with high N-delivery efficiency. Urea represents a particularly efficient N source, yielding two NH_4_^+^ per hydrolyzed molecule and delivering N more efficiently than NH_4_^+^, NO_3_^−^ (*25*) and some amino acids such as leucine and glutamate (*26*). This biochemical advantage, combined with leucine’s negligible contribution to ambient N pools (0.22%), suggests that urea plays a more important role in supporting *Synechococcus* growth in nutrient-depleted environments. Consistent with this, previous studies in the Atlantic Ocean have shown that *Synechococcus* outcompetes other microorganisms (heterotrophic bacteria, eukaryotic phytoplankton, and *Prochlorococcus*) for urea uptake but is comparatively less competitive for leucine acquisition (*8*). Experimental evidence further confirms that urea stimulates the growth of *Synechococcus* more effectively than inorganic N (NH_4_^+^ or NO_3_^−^) under nutrient-depleted conditions (*10*).

In nutrient-rich waters, *Synechococcus* exhibited higher glucose uptake activity (60% versus 24%), rates (*P* = 9.06 × 10^−25^), and glucose-derived C_net_% (*P* = 6.56 × 10^−10^) (Fig. 1 and tables S7 and S8), likely reflecting the increased substrate availability for high cellular energy demand. Glucose serves primarily as an energy source, as its extracellular uptake bypasses the ∼18 ATP required for de novo glucose biosynthesis (*27*). Nutrient-rich *Synechococcus* possesses extensive sensor-response systems, such as histidine kinase sensors and associated response regulators (*21*), whose autophosphorylation requires ATP expenditure (*28*). This elevated energy demand, together with higher glucose concentrations in nutrient-rich waters, facilitate uptake via both active transport and passive diffusion (*29*), likely driving increased glucose assimilation in nutrient-rich populations. Note that because part of the imported glucose may be diverted to energy metabolism and respiration (*30*, *31*), the glucose uptake measured here represents net rather than gross glucose uptake and thus may underestimate its contribution to cellular carbon acquisition.

The lineage-specific mixotrophic phenotype underlies the trophic adaptations of *Synechococcus* (*32*), yet whether this trait is plastic or evolutionary fixed remains poorly resolved. If lineage-specific mixotrophic phenotypes persist across environmental conditions, then this stability would suggest evolutionary adaptation to their native habitats (*33*). To address this, we quantified substrate uptake in laboratory-cultured strains representing the dominant nutrient-depleted and nutrient-rich clades from our field sites (fig. S1B). Despite long-term growth under identical nutrient-replete conditions, nutrient-depleted strains consistently showed higher urea uptake and urea-derived N_net_%, whereas nutrient-rich strains showed higher glucose uptake and glucose-derived C_net_% (*P* < 0.001 for all comparisons; Fig. 2 and table S13). These results indicate that these mixotrophic traits are stable, lineage-encoded adaptations rather than short-term plastic responses, shaped by and facilitating fitness within the nutrient regimes of their native habitats (*4*).

This lineage-specific differentiation was also evident in the broader context of heterotrophic organic matter uptake (figs. S4 and S5, and tables S9 and S10) despite the known exchange of inorganic and organic matter between *Synechococcus* and heterotrophs (*34*). Given their high numerical abundance (table S1), heterotrophic bacteria likely represent a major sink for organic substrates (*35*, *36*). At the single-cell level, however, nutrient-depleted *Synechococcus* showed markedly higher urea uptake and N_net_% than heterotrophic bacteria, highlighting their specialized capacity for urea acquisition. For glucose, heterotrophic bacteria generally showed higher uptake rates and glucose-derived C_net_% than *Synechococcus*, particularly in nutrient-rich waters. Nonetheless, *Synechococcus* from nutrient-rich waters still maintained higher glucose uptake and C_net_% compared to those in nutrient-depleted waters despite the intensified competition. Together, these comparisons indicate that nutrient-depleted and nutrient-rich *Synechococcus* express distinct, lineage-specific patterns of organic substrate use, even against a background of substantial heterotrophic organic matter uptake.

### Genetic drivers of contrasting organic nutrient utilization between nutrient-depleted and nutrient-rich *Synechococcus*

Intrinsic phenotypes are shaped by underlying genetic variation (*37*). Corresponding to the observed elevated urea uptake in nutrient-depleted waters, *Synechococcus* exhibited up-regulation of urea transporters, with expression exceeding that of other N transporters (e.g., amino acids, inorganic N, and cyanate) (Fig. 3, A and B), indicating a greater transcriptional investment in urea acquisition relative to the eutrophic site. The analysis of global Tara Oceans datasets further revealed that this metabolic potential is pervasive and tightly linked to ambient inorganic N availability. The proportional contribution of urea transporters to total N transporter abundance and expression was negatively correlated with ambient NO_3_^−^ concentration (*P* = 6 × 10^−10^ and 8.2 × 10^−09^; Fig. 3, C and D), demonstrating enhanced transport potential for urea under N-depleted conditions (*38*). In addition, the potential for transporting another important organic N source, cyanate (*39*), was notably high at our nutrient-depleted site and in global low-N regions (NO_3_^−^ = 0 to 2 μM), with particularly elevated *cynABD* expression in the N-limited Indian Ocean and Mediterranean Sea (fig. S8) (*40*, *41*). This site-specific urea and cyanate transport potential was further exemplified by clade III, which was exclusive to the nutrient-depleted site and exhibited higher cell-normalized expression than the more abundant clade II (figs. S1 and S9). Combined with previous reports of enhanced transport potential for nitriles and cyanides in low-N regions, these findings collectively suggest that nutrient-depleted *Synechococcus* broadly exhibit metabolic potential for multiple organic N substrates (*32*, *42*), supporting a widespread adaptive capacity for organic N acquisition in nutrient-depleted environments.

Despite low ambient urea concentrations at the nutrient-depleted site (table S1), *Synechococcus* achieved higher absolute uptake rates than nutrient-rich populations. Classical microbial uptake kinetics predict that low substrate concentration would constrain assimilation (*43*), yet nutrient-depleted *Synechococcus* appear to surmount this limitation via high-affinity transporters, analogous to nutrient-depleted specialists such as SAR11 (*44*). The stable expression of the urease operon across different nutrient conditions (*P* = 0.12; fig. S6) indicates that transport capacity, rather than downstream enzymatic hydrolysis, is the key determinant of urea utilization efficiency. Supporting this, the high-affinity urea transporter *DUR3* (*17*) showed significantly elevated expression at the nutrient-depleted site and in global low-N regions (*P* = 0.02 and 0.002; Fig. 4), suggesting that it plays a critical role in enabling efficient N acquisition under extreme nutrient scarcity (*45*). Furthermore, the higher investment of high-affinity branched-chain amino acid transporter (*livFGHKM*) (*46*) at our nutrient-depleted site (Fig. 3B), despite global variability (fig. S7), suggests that high-affinity transport may represent a lineage-specific adaptation to nutrient scarcity.

Similarly, the higher glucose uptake rates observed in nutrient-rich *Synechococcus* (*P* = 9.06 × 10^−25^; Fig. 1) were partially driven by the use of distinct transporters under different nutrient regimes (fig. S10). In both the nutrient-rich site and global high-N regions (NO_3_^−^ > 2 μM), *Synechococcus* had more copies and expressed higher levels of the sugar/H^+^ symporter permease *glcP* compared with nutrient-depleted populations (fig. S10, E to H). Unlike the high-affinity but energy-intensive *glcH* (*47*), permease *glcP* uses the proton motive force rather than direct ATP hydrolysis, facilitating rapid and efficient glucose uptake when ambient concentrations are elevated (*48*). Thus, this lower direct ATP cost, coupled with the higher energy demands of nutrient-rich waters, likely favors the preferential expression of *glcP* for more efficient glucose turnover. This pattern suggests that nutrient-rich *Synechococcus* may rely on the energetically favorable transporter to meet their energy demand under nutrient-rich conditions. Collectively, these genetic patterns underpin distinct urea and glucose uptake between nutrient-depleted and nutrient-rich *Synechococcus*, revealing environment-driven divergence in organic nutrient utilization.

### Undervalued role for organic nitrogen in *Synechococcus* nutrition and primary production

Our quantitative estimates demonstrate that organic N represents a substantial nutritional supplement for nutrient-depleted *Synechococcus*. At the nutrient-depleted site, organic N from urea and leucine, predominantly urea, constituted 40 to 63% of estimated total N uptake, which is approximately fourfold higher than in nutrient-rich waters (8 to 15%) (Fig. 5). Moreover, multiple lines of evidence further underscore the ecological and competitive importance of different organic N substrates for *Synechococcus* in low-nutrient regimes, including higher urea uptake rates of *Synechococcus* relative to *Prochlorococcus*, picoeukaryotes, and heterotrophic bacteria (*8*, *9*), as well as their dominance of cyanase expression across the nonpolar surface ocean (*49*).

Using lineage-specific C:N cellular stoichiometry (nutrient-depleted: 6.0 to 9.5; nutrient-rich: 4.1 to 7.9; table S4), we further estimated that amended organic N substrates support 42 to 66% of primary production in nutrient-depleted waters but only 9 to 15% in nutrient-rich waters (Fig. 5). Given the additional unmeasured organic N compounds present in marine systems [e.g., cyanate, chitin, and glutamate (*42*, *49*, *50*)], these estimates likely underestimate the realistic contribution of organic N to *Synechococcus* productivity. Beyond nutrition, urea also enhances acid-stress tolerance (*51*) and increases RuBisCO activity and biosynthesis of sugars and proteins under warming conditions (*52*), suggesting broader physiological benefits. These benefits likely extend to other phytoplankton as well, since many groups [e.g., diatoms (*53*) and dinoflagellates (*54*)] can use multiple forms of organic N (*45*).

Despite increasing evidence for the importance of organic N, current marine biogeochemical and ecosystem models typically simplify total N uptake as purely inorganic. This simplification limits the accuracy of forecasts regarding future phytoplankton abundance (*55*), net community production (*56*), and inorganic N limitation effects on productivity (*57*). Considering the undervalued ecological and physiological importance of organic N acquisition, incorporating lineage-specific organic N utilization into these models is essential for accurately predicting marine productivity and niche partitioning under both present and future conditions. This need is particularly urgent in a warming, stratifying ocean, where organic N may play an increasingly central role.

### Study limitations

While our single-cell analyses provide high-resolution insights into *Synechococcus* mixotrophy, certain quantitative interpretations, specifically the estimated contributions of organic substrates to total N and C acquisition, rely on necessary biogeochemical assumptions. These include the application of literature-derived elemental stoichiometry (C:N ratios), urea concentrations below analytical detection limits (table S1), and standard correction factors for tracer-based assimilation (table S3). Accordingly, these calculated contributions should be viewed as representative semi-quantitative estimates of metabolic niches, rather than as direct measurements with fully constrained uncertainty. Furthermore, while independent biological triplicates were used in the incubation experiments, cells were pooled before NanoSIMS analysis to ensure sufficient cell density for high-resolution imaging (*58*). Consequently, the resulting single-cell distributions reflect composite population-level patterns rather than bottle-specific variance. While the large number of analyzed cells effectively captures phenotypic heterogeneity, it simultaneously increases statistical sensitivity to subtle variations. Future research incorporating broader spatial replication, independent replicate-level NanoSIMS analyses, and direct quantification of ambient dissolved organic pools will be essential to further refine these metabolic estimates and their associated uncertainty propagation.

## MATERIALS AND METHODS

### Field sampling

Field experiments were conducted in the nutrient-depleted South China Sea (stations A and B) and nutrient-rich coastal waters adjacent to Xiamen Island (station C) in June 2022 and June 2023, respectively (fig. S1A). At each site, surface seawater samples (2- to 5-m depth) were collected and prefiltered through a 20-μm nylon mesh to remove large organisms and particles. Biogeochemical samples were collected in triplicate and analyzed following standard procedures (Supplementary Methods), including abundances of *Synechococcus* and heterotrophic bacteria, concentrations of dissolved inorganic carbon, inorganic nutrient (NO_3_^−^, NO_2_^−^, NH_4_^+^, and PO_4_^3−^), urea, leucine, and DOC. For DNA and RNA extraction, 20 liters of prefiltered seawater was collected in triplicate on 0.22-μm polycarbonate filters (142 mm; Millipore, USA). Filters were flash-frozen in liquid nitrogen and stored at −80°C until nucleic acid extraction. No permit or specific permission was required to collect the environmental seawater samples used in this manuscript.

### Laboratory cultivation of *Synechococcus* strains

Five *Synechococcus* strains—representing nutrient-depleted (strain YX04-3-clade III, WH8012-clade II) and nutrient-rich (strain CC9311-clade I, XM-24, and XM-11-clade CB5) strains (fig. S2)—were selected for laboratory cultivation. Strains were cultured in SN medium (*59*) prepared using 0.22-μm filtered natural seawater from the Xiamen coast. Cultures were maintained in a shaker at 22°C under constant light flux with ∼30 μmol photons m^−2^ s^−1^. To mitigate the suppressive effects of replete inorganic nutrients on organic matter uptake, cultures in SN medium (40 ml) underwent four cycles of threefold dilution with sterile natural seawater at the onset of each exponential phase, which gradually reduced nutrient concentrations and yielded a final volume of ∼3 liters. The diluted cultures were then prepared for stable isotope tracer incubations. Biogeochemical samples from diluted cultures were collected at the beginning of the incubation and measured in triplicate following the same procedures as field samples (Supplementary Methods).

### Experimental setup

The field incubation experiments were performed using the following procedures. Prefiltered seawater from each station was aliquoted into 12 acid-cleaned 2-liter polycarbonate bottles, with 1.2 liters per bottle. All bottles were acid-cleaned by soaking in 10% HCl for 2 days and rinsing five times with Milli-Q water. The 12 bottles were divided into two groups to avoid isotopic overlap among different substrates. Group one (six bottles) was amended with NaH^13^CO_3_ (99%; Cambridge, MA, USA) and ^15^N-leucine (98%; Cambridge, MA, USA), and group two (six bottles) was amended with ^13^C-glucose (99%; Cambridge, MA, USA) and ^15^N-urea (98%; Sigma-Aldrich, USA). All isotopes (except for ^15^N-leucine) were added as 10 to 50% of estimated ambient concentrations, and their final concentrations are provided in table S3.

Following isotope addition, triplicate bottles from each group (control) were immediately pooled (serving as the initial control; table S2 summarizes the coefficients of variation for biochemical parameters across replicates at each station). The pooled samples were then filtered through 0.22-μm acid-cleaned polycarbonate filters (47 mm; Millipore, USA). The samples were concentrated to ∼30 ml to determine the initial ^13^C and ^15^N isotopic abundance. The remaining three bottles in each group (experimental) were incubated in an on-deck incubator reproducing the light intensity and temperature at sea surface through continuous circulation of surface seawater on deck. All incubations were started at about 12:00 hours and lasted for 3 hours to minimize isotopic exchange between *Synechococcus* and coexisting bacteria (*60*). Incubations were stopped by concentrating seawater to ∼30 ml as described above. The concentrated samples were fixed with 0.5% (v/v) glutaraldehyde in darkness for 30 min, then flash-frozen in liquid nitrogen, and stored at −80°C until cell sorting.

Laboratory incubations of diluted cultures of each *Synechococcus* strain were conducted following procedures similar to the field experiments, except that the cultures were incubated in 250-ml polycarbonate bottles (125 ml per bottle), placed under their original growth conditions, and incubated for 2 hours.

### Cell sorting, NanoSIMS sample preparation, and analysis

Cells of *Synechococcus* and heterotrophic bacteria were sorted using a BD FACSAria flow cytometer with a 488-nm excitation laser and a 70-μm nozzle (fig. S3A). The sorted cells were dehydrated on membranes using ethanol and air-dried until NanoSIMS analysis. The air-dried membranes were analyzed using a NanoSIMS 50 L (CAMECA-Ametek, Gennevilliers, France) in Academia Sinica, Taiwan. Heterotrophic bacteria were analyzed only from the representative stations (sites B and C) and the representative laboratory cultures of *Synechococcus* strains YX04-3 and XM-24. To validate sorting reliability, additional *Synechococcus* cells were sorted for internal transcribed spacer (ITS) sequencing. As a relatively large number of cells were required, we selected only nutrient-depleted site as representative examples to validate the sorting accuracy. Details are provided in Supplementary Methods.

### Rate, X_net_%, and contribution calculations

The isotope percent abundances of ^13^C and ^15^N were calculated according to the following equations

AC13=C12C−132(C2−12+C12C−13)×100% (1)

AN15=C12N−15(C12N−14+C12N−15)×100% (2)

In which ^12^C_2_^−^, ^12^C^13^C^−^, ^12^C^14^N^−^, and ^12^C^15^N^−^ are total counts of secondary ions of ^12^C_2_^−^, ^12^C^13^C^−^, ^12^C^14^N^−^, and ^12^C^15^N^−^, respectively.

Single-cell C- or N-specific uptake rates (V, amol C cell^−1^ hour^−1^ or amol N cell^−1^ hour^−1^) were calculated using Eq. 3 (*8*). Because of the low ambient concentrations, rates for urea and glucose were corrected to reduce potential stimulation effects on cellular uptake induced by excessive isotopic tracer addition (Supplementary Methods) (*9*). The correction factors for each station and laboratory cultures are provided in table S3

$$\text{Cell}-\text{specific uptake rate}\;\left(V\right)=\frac{A_{\text{cell}}-\bar{A_{t0}}}{A_{\text{source}}-\bar{A_{t0}}}\times \frac{1}{t}\times Q_{\text{cell}}$$ (3)

where $A_{\text{cell}}$, $\bar{A_{t0}}$, and $A_{\text{source}}$ are the isotopic percent abundances (AC13 or AN15) of the cells after incubation, of the cells before incubation (mean value), and of the source pool, with *t* as the incubation time. $Q_{\text{cell}}$ is the estimated C and N cell contents that are calculated on the basis of cell biovolume (BV), assuming that the cells are spherical (*61*–*63*). $Q_{\text{cell}}$ for *Synechococcus* ($C_{\text{Syn}}$ and $N_{\text{Syn}}$) and heterotrophic bacteria ($C_{H}$ and $N_{H}$) was calculated as Eqs. 5 and 6 and Eqs. 7 and 8, respectively

$$\text{BV}=\frac{4}{3}\pi r^{3}$$ (4)

$$C_{\text{Syn}}\;\left(\text{pg}/{\mathrm{\mu m}}^{3}\right)=0.433\times {\text{BV}}^{0.863}$$ (5)

$$N_{\text{Syn}}\;\left(\text{pg}/{\mathrm{\mu m}}^{3}\right)=0.083\times BV^{0.837}$$ (6)

$$C_{H}\;\left(\text{pg}/{\mathrm{\mu m}}^{3}\right)=0.121\times \text{BV}$$ (7)

$$N_{H}\;\left(\text{pg}/{\mathrm{\mu m}}^{3}\right)=0.121\text{BV}/17.228\times 10^{-3.153\text{BV}}$$ (8)

Cells were considered active when the percent abundance enrichment ($A_{\text{cell}}-\bar{A_{t0}}$) was higher than the SD of the cellular percent abundances. A total of 4836 *Synechococcus* cells and 1458 heterotrophic bacterial cells were detected (the total and active cell numbers for each sample were summarized in tables S7 and S9). The proportion of active cells was calculated as the ratio of active cells to the total number of cells per sample.

Net assimilation percentages (X_net_%) were calculated using Eq. 9

$$X_{\text{net}}\%=\frac{A_{\text{cell}}-\bar{A_{t0}}}{A_{\text{source}}-\bar{A_{t0}}}\times 100$$ (9)

where $X_{\text{net}}\%$ represents the fraction of the total cellular C or N pool derived from substrate assimilated during the incubation period. This expression is mathematically equivalent to previously published formulations (*64*, *65*) but is written here in the isotopic-abundance form used in our calculations.

The cell-specific contributions of amended organic substrates to estimated total C uptake (OC%) and total N uptake (ON%) were calculated as follows

$$\text{OC}\%=\frac{V_{\text{glucose}-C}+V_{\text{leucine}-C}+V_{\text{urea}-C}}{V_{\text{NaHCO}3-C}+V_{\text{glucose}-C}+V_{\text{leucine}-C}+V_{\text{urea}-C}}\times 100$$ (10)

$$V_{N-\text{estimated total nitrogen}}=\frac{V_{\text{NaHCO}3-C}+V_{\text{glucose}-C}+V_{\text{leucine}-C}+V_{\text{urea}-C}}{C:N\;\text{molar ratio}}$$ (11)

$$\mathrm{ON}\%=\frac{V_{\text{leucine}-N}+V_{\text{urea}-N}}{V_{\text{estimated total}\;N}}\times 100$$ (12)

where $V_{\text{NaHCO}3-C}$, $V_{\text{glucose}-C}$, $V_{\text{leucine}-C}$, $V_{\text{urea}-C}$, $V_{\text{leucine}-N}$, and $V_{\text{urea}-N}$ are C or N uptake rates from the corresponding substrates. Total C uptake was calculated as the sum of $V_{\text{NaHCO}3-C}$, $V_{\text{glucose}-C}$, $V_{\text{leucine}-C}$, and $V_{\text{urea}-C}$. Total N uptake ($V_{\text{estimated total}\;N}$) was estimated by dividing total C uptake by the lineage-specific cellular C:N molar ratio, assuming proportionality between total cellular C and N uptake (*66*–*69*). Specifically, C:N ratios of 6.0 to 9.5 and 4.1 to 7.9 were applied to the nutrient-depleted and nutrient-rich lineages, respectively (summary of C:N ratios in table S4).

### *Synechococcus* genome collection and gene calling

To construct reference gene sets for added substrate utilization in *Synechococcus*, we obtained 655 genomes with completeness >70 and < 10% contamination from the Global Ocean Microbiome Genome Catalogue (*70*), Cyanorak (https://cyanorak.sb-roscoff.fr/cyanorak), the National Center for Biotechnology Information (NCBI) database (www.ncbi.nlm.nih.gov/datasets/genome/?taxon=1129), and the Ocean Microbiomics database (https://microbiomics.io), as of December 2024. In addition, four metagenome-assembled genomes (MAGs) recovered from China estuaries and 10 genomes of isolates were included in the analysis. Genome quality was assessed using CheckM v1.2.2 (*71*), and the qualified genomes are listed in table S5. A phylogenetic tree was constructed to validate clade classification of the five laboratory-studied strains using 24 nearly complete reference *Synechococcus* genomes (details in Supplementary Methods).

Protein-coding genes were predicted using Prodigal v2.6.3 (*72*) to generate protein files. These files were then used to identify single-copy genes (*73*) and genes involved in N substrate utilization and glucose transport using HMMER v3.4 (*e* value < 1 × 10^−20^) or BLASTp (coverage > 50%, identity > 50%, and *e* value < 1 × 10^−10^) (reference gene list in table S6). For *glcH*, which lacks a defined HMM profile, the reference sequences were downloaded from UniProt (*74*), while sequences for other putative glucose transporters were retrieved from Transporter Classification Database (2024) (*75*). Candidate sequences of each gene were validated against the NCBI non-redundant (nr) database and then clustered using CD-HIT v4.8.1 (*76*). Representative sequences from each gene cluster were then used as their reference gene sets (details in Supplementary Methods).

### ITS, metagenomic and metatranscriptomic sequencing, and bioinformatic analysis

The DNA and RNA from in situ seawater and DNA of sorted *Synechococcus* cells were extracted using the ZymoBIOMICS DNA/RNA Miniprep Kit (ZYMO). To characterize field and sorted *Synechococcus* populations, the ITS region was amplified from DNA samples with primers 16*S* (5′-TGGATCACCTCCTAACAGGG-3′) and 23*S* (5′-CCTTCATCGCCTCTGTGCC-3′) (*77*). The SMRTbell prep kit 3.0 was used to obtain the libraries for Pacbio sequel IIe sequencing. Raw sequences were quality controlled, trimmed, and then dereplicated using Mothur v.1.48.1 (*78*). Dereplicated representative sequences were subsequently classified with *Synechococcus* ITS sequence classification tool (Syn-Tool) (*79*). The nutrient-depleted sites were dominated by clade II (94%) and clade III (5%), whereas the nutrient-rich site exhibited higher clade diversity, including clade II (67%), clades CB5 and CB4 (22%), and clade IX (9%) (fig. S1B). Sorted *Synechococcus* populations closely matched the in situ clade composition (fig. S3B), confirming the reliability of the cell sorting procedure.

Remaining quality-approved DNA samples were subjected to library preparation using the VAHTS Universal Pro DNA Library Prep Kit following the instructions. RNA samples were processed into sequencing libraries using Ribo-clean rRNA Depletion Kit Mega (Bacteria)–RN417 following the manufacturer’s recommendations. Both libraries were sequenced on the Illumina MGI2000 platform using PE150 sequencing. Raw metagenomic sequencing reads were quality controlled using the read_qc module from metaWRAP v1.3.2 (*80*). Raw metatranscriptomic data were processed with FASTP v0.23.0, filtering out reads with a mean quality score < 20 or a length < 75 base pairs (*81*). All rRNA reads were removed using SortMeRNA v.4.3.7 with default settings (*82*). Clean metagenomic and metatranscriptomic reads were then mapped to the reference gene sets of *Synechococcus* using bowtie2 v2.33 (-k 1 to output the top hits) (*83*). Read counts and gene lengths were calculated using coverM v0.3.2 (identity > 90% and coverage > 50%) (*84*) and then used to calculate gene or transcript abundance as transcripts per million (TPM) (*85*). For intersample comparison, gene and transcript abundance were normalized to median gene [single-copy marker gene (SCMG)] and transcript [single-copy marker transcript (SCMT)] abundance of 10 single-copy marker genes, respectively (relative abundance = gene abundance/SCMG abundance, relative expression = transcript abundance/SCMT abundance). To compare the investment in different N acquisition pathways, the relative abundance and expression of N substrate transport genes (including amino acids, urea, cyanate, and inorganic N) were calculated as fractional contributions (ranging from 0 to 1), where the sum of all targeted transporters equals 1. These normalized values were then visualized using radar charts generated with the R package fmsb v0.7.6 in RStudio.

### Gene abundance and expression patterns in the global ocean

To investigate the global abundance and expression of the N substrate and glucose transporters in *Synechococcus*, we queried the *Tara* Oceans metagenomic and metatranscriptomic database using the online webserver Ocean Gene Atlas v2.0 (*86*). HMM files generated from our custom reference gene sets were used as queries to identify homologous sequences within Ocean Gene Atlas. The search results were filtered by an *e* value threshold < 1 × 10^−40^. To minimize the potential effect of light availability on gene expression, we restricted our analysis to *Synechococcus* hits from the surface and deep chlorophyll maximum layers.

The abundance of each gene and transcript was normalized to the median abundance of *Synechococcus* SCMGs and SCMTs, respectively (*87*). Normalized gene relative abundance or expression was used to calculate the contribution of urea transporters to the summed abundance or expression of different N substrate transporters. The relationship between urea transporter contribution and ambient nitrate (NO_3_^−^) concentration was visualized with ggplot2 v3.5.2 in RStudio.

### Statistical analysis

Differences in single-cell C and N uptake rates were assessed using the nonparametric Wilcoxon test in RStudio. Specifically, uptake rates were compared between nutrient-depleted and nutrient-rich sites, between nutrient-depleted and nutrient-rich *Synechococcus* strains, and between *Synechococcus* and heterotrophic bacteria. The Wilcoxon test was also applied to assess differences in gene relative abundance and expression between nutrient-depleted and nutrient-rich sites and between high-N and low-N regions. Exact *P* values and sample sizes (*n*) of all comparisons were reported in table S13.

## Supplementary Materials

### The PDF file includes:

Supplementary Methods Figs. S1 to S10 Legends for tables S1 to S13 Legend for data S1 References
