# Supplementary material for: Single-cell quantification reveals divergent mixotrophic strategies underlying niche partitioning in marine Synechococcus
Source: Sci Adv. 2026 Aug 21;12(34):eaef8347. doi: 10.1126/sciadv.aef8347 (PMC13505874; doi:10.1126/sciadv.aef8347)
Supplement: Supplementary file 1 [file sciadv.aef8347_sm.pdf]

Science Advances
AAAS

Supplementary Materials for

Single-cell quantification reveals divergent mixotrophic strategies underlying niche partitioning in marine *Synechococcus*

Bowen He *et al.*

Corresponding author: Qiang Zheng, zhengqiang@xmu.edu.cn

Sci. Adv. **12**, eaef8347 (2026)
DOI: 10.1126/sciadv.aef8347

The PDF file includes:

Supplementary Methods
Figs. S1 to S10
Legends for tables S1 to S13
Legend for data S1
References

Other Supplementary Material for this manuscript includes the following:

Tables S1 to S13
Data S1

Supplementary Methods

Biogeochemical sampling

Samples for *Synechococcus* and heterotrophic bacteria enumeration (2 mL per sample) were fixed with 0.5% glutaraldehyde (30 min, dark) and stored at −80°C. Triplicate seawater samples (200 mL per sample) were filtered for chemical parameter analysis: 100 mL of seawater were filtered through a 0.22 μm polycarbonate membrane (47 mm, Millipore, USA). 20 mL filtrates were collected and preserved with 0.02% saturated $HgCl_2$ solution for dissolved inorganic carbon (DIC). 45 mL filtrates were collected in polypropylene tubes for inorganic nutrients. The remaining 100 mL seawater were filtered through 0.7 μm GF/F glass fiber membranes (25 mm, Whatman, UK). Filtrates for urea (30 mL per sample), leucine (1.5 mL per sample), and dissolved organic carbon (DOC; 25 mL per sample) were collected in glass vials (CNW, Shanghai, China). Samples for inorganic and organic nutrients were stored at −20°C. The tubes and vials were acid cleaned by soaking in 10% HCl for 2 days and then rinsing 5 times with Milli-Q water. The GF/F and acid-cleaned vials were precombusted at 450 °C for 4 h.

Biogeochemical analysis

Measurement of Synechococcus and heterotrophic bacteria abundances

Synechococcus and heterotrophic bacteria abundances were measured using a BD FACSAria^{TM} flow cytometer equipped with a 488 nm excitation laser. Heterotrophic bacteria

samples were stained using SYBR Green I staining (Invitrogen, Carlsbad, CA, USA) in darkness for 15 minutes prior to injection. *Synechococcus* were differentiated in unstained samples based on autofluorescence phycoerythrin (575 ± 26 nm, orange), chlorophyll (695 ± 40 nm, red), and forward scattered light (size), while Fluorescein isothiocyanate (FITC) fluorescence (530 ± 30 nm) and chlorophyll were used to distinguish stained heterotrophic bacteria. Fluorescent beads (1 μm) were added as internal standards. Cell enumeration was conducted using the BD FACSAria^{TM} Software.

Measurement of chemical parameters

Dissolved inorganic carbon (DIC) was measured using a DIC analyzer (AS-C3, Apollo SciTech, USA) with a precision of ± 0.1%. The instrument was calibrated to an accuracy of ± 2 μM using certified reference materials (Batch #207, A.G.). Inorganic nutrient concentrations were determined by a SEAL AA500 Auto Analyzer (Seal Analytical, US) with standard photometric methods. Urea concentrations were measured using a five-centimeter cuvette based on the optimized colorimetric reaction with diacetyl monoxime (*88*). Since measured values in the South China Sea were below the detection limit (100 nM), we adopted the median concentration (90 nM) reported by Chen et al. (2014) (*88*). Free leucine concentrations were determined using an ultrahigh performance liquid chromatography instrument (Agilent 1260) equipped with a fluorescence detector (*89*). DOC samples were defrosted and acidified to pH 2–3 with 2 N hydrochloric acid, and then measured by high-temperature catalytic oxidation method

using a Shimadzu total organic carbon (TOC) analyzer. Glucose concentration was estimated as 0.2% of the DOC concentration in the nutrient-depleted site and 0.4% of the DOC concentration in the nutrient-rich site and laboratory cultures (*90–92*).

Flow cytometry cell sorting

Cells were sorted using a BD FACSAria™ flow cytometer with a 488 nm excitation laser and a 70 μm nozzle, using BD FACSFlow™ Sheath Fluid. Prior to sorting, the instrument was sterilized through the "Prepare for Aseptic Sort" protocol, followed by drop delay calibration using BD™ Accudrop Fluorescent Beads. All sorts were run in "purity" mode at a flow rate of 1, with sorting efficiency (> 95%) verified by counting a specific number of sorted yellow–green microspheres (1 μm) under fluorescence microscopy. Sort gates for *Synechococcus* were established based on phycoerythrin, chlorophyll, and forward scattered light (Fig. S3A). Heterotrophic bacteria samples were stained with SYBR Green I (Invitrogen, Carlsbad, CA, USA) for 15 minutes in darkness and then distinguished using FITC and chlorophyll (Fig. S3A). For each population, approximately 10^6 sorted cells were filtered onto a 0.22 μm Au-Pd precoated polycarbonate membrane. The cells were subsequently dehydrated through a graded ethanol series (100 μL of 30%, 50%, 70%, 90%, and 100% ethanol), followed by a final 2-min soak in absolute ethanol. Membranes were dried on glass slides and stored in a dark and dry environment at room temperature until nanoscale secondary ion mass spectrometry (NanoSIMS) analysis. To validate sorting reliability, an additional ~ 10^7 sorted *Synechococcus* cells from the

nutrient-depleted site were collected on 0.22 μm polycarbonate filters for ITS sequencing.

NanoSIMS analysis

The air-dried membranes were analyzed using a NanoSIMS 50 L (CAMECA-Ametek, Gennevilliers, France) in Academia Sinica, Taiwan. Each area of 35 × 35 to 50 × 50 μm^2 was presputtered by a 704 to 880 pA Cs^+ primary beam for 3 min and analyzed by 10.9-13.8 pA Cs^+ primary beam for 13 to 44 min over multiple areas of each membrane sample to collect image data with a lateral resolution of 400-500 nm. The pixel dwell time was 1 millisecond. Secondary ions ($^{16}O^-$, $^{12}C_2^-$, $^{12}C^{13}C^-$, $^{12}C^{14}N^-$, $^{12}C^{15}N^-$, $^{28}Si^-$, and $^{32}S^-$) were simultaneously collected by multiple electron multipliers (EMs) with a 30 μm entrance slit and a 200 μm aperture slit, providing a mass resolving power of > 9000(M/△M). The pulse height distribution of EMs was adjusted every 3-6 image acquisitions to keep a constant signal efficiency. All data were processed using the LIMAGE software (developed by Larry Nittler, Arizona State University, USA). Cell regions were outlined in $^{12}C^{14}N^-$ ion images by the particle finding algorithm in LIMAGE. The data for each cell contain more than 3×10^5 $^{12}C^{14}N^-$ counts. Poor-quality data were manually excluded based on cellular shape and size.

Correction of cell-specific uptake rates

The addition of isotope-labeled tracers (^{13}C-glucose and ^{15}N-urea) was more than 30% of the initial pool (Table S1 and S3). In order to account for a potential uptake stimulation, cell-specific

uptake rates were corrected according to Berthelot et al. (2019) (*9*), based on Rees et al. (1999) (*93*):

$$\text{uptake corrected} = \frac{\text{original uptake}}{\frac{\text{Conc}_{\text{incub}}}{\text{Ks} + \text{C}_{\text{incub}}} * \frac{\text{Ks} + \text{Conc}_{\text{Amb}}}{\text{Conc}_{\text{Amb}}}}$$

$Conc_{incub}$ is the sum of ambient and final tracer concentrations. $Conc_{Amb}$ represents the ambient concentration and Ks is the half-saturation parameter assuming Michaelis-Menten kinetics. The K_s values of glucose (21.55 nM) and urea (20 nM) were referred from Muñoz et al. (2017) and Sahlsten et al. (1987) (*47*, *94*). The correction factors reduced C or N uptake rates by an average factor of 1.072 for glucose and 1.058 for urea, respectively (Table S3). These adjustments were uniformly applied across different populations, assuming comparable kinetic constants.

***Synechococcus* genome collection, phylogenomic tree and reference gene extraction**

A total of 1010 *Synechococcus* genomes, including four MAGs and ten self-sequenced genomes, were qualified using CheckM v1.2.2 (https://github.com/Ecogenomics/CheckM/wiki), retaining 669 genomes with > 70% completeness and < 10% contamination. These qualified genomes were processed through Prodigal v2.6.3 for gene prediction (*72*) and annotated using PROKKA v1.11 (*95*). To assign these genomes to specific clades, average nucleotide identity (ANI) was calculated for all 669 genomes using FastANI v1.33. Genomes were clustered using a

95% ANI threshold, and taxonomic assignments were made based on their clustering with reference genomes of known clades. For genomes that remained unclassified via ANI, internal transcribed spacer (ITS) sequences were extracted using barrnap v0.9 (https://github.com/tseemann/barrnap) and compared against a comprehensive *Synechococcus* ITS database (*79*) using BLASTn v2.11.0 to assign their clade identity (identity > 95%, coverage > 80%, and e-value < 1E-10). Finally, genomes of the five studied *Synechococcus* strains were aligned with 24 nearly completed reference *Synechococcus* strains to assess phylogenetic relatedness using GTDB-TK (GTDB release R202). The alignment file was used to build the phylogenetic tree using IQTREE v 2.2.6 (*96*), with ModelFinder (*97*) to select the best phylogenetic model. The tree was subsequently visualized using Chiplot (*98*).

For single-copy and N-related gene identification, HMMs were downloaded from the KO.hmm database (*99*) (all used HMM files are listed in Data S1). Candidate gene sets for each gene were identified by searching predicted protein sequences (faa files) against these HMM profiles using HMMER v3.4 (e-value < 1E-20) (*100*). Putative glucose transporters were identified using three complementary approaches: (i) BLASTp searches against *Synechococcus* *glcH* reference proteins (*101*) retrieved from UniProt (*74*), applying coverage > 50%, identity > 50%, and an e-value < 1E-10; (ii) HMMER v3.4 searches for the *glcP* permease; and (iii) DIAMOND v2.0.6 searches against the Transporter Classification Database (TCDB, 2025) (*75*) in sensitive mode with an e-value cutoff of 1E-5 to select the best-scoring hits. All candidate

gene sets were subsequently validated against the NCBI nr database (accessed February 25th, 2025, including Subject Taxonomy IDs) with e-value < 1E-10 and coverage > 50% cutoffs, followed by manual annotation verification. Validated protein sequences were clustered using CD-HIT v4.8.1 at 95% identity and 90% coverage (*76*). Representative protein sequences were used to retrieve their corresponding coding nucleotide sequences from ffn files, generating the final reference gene sets.

Supplementary Figures

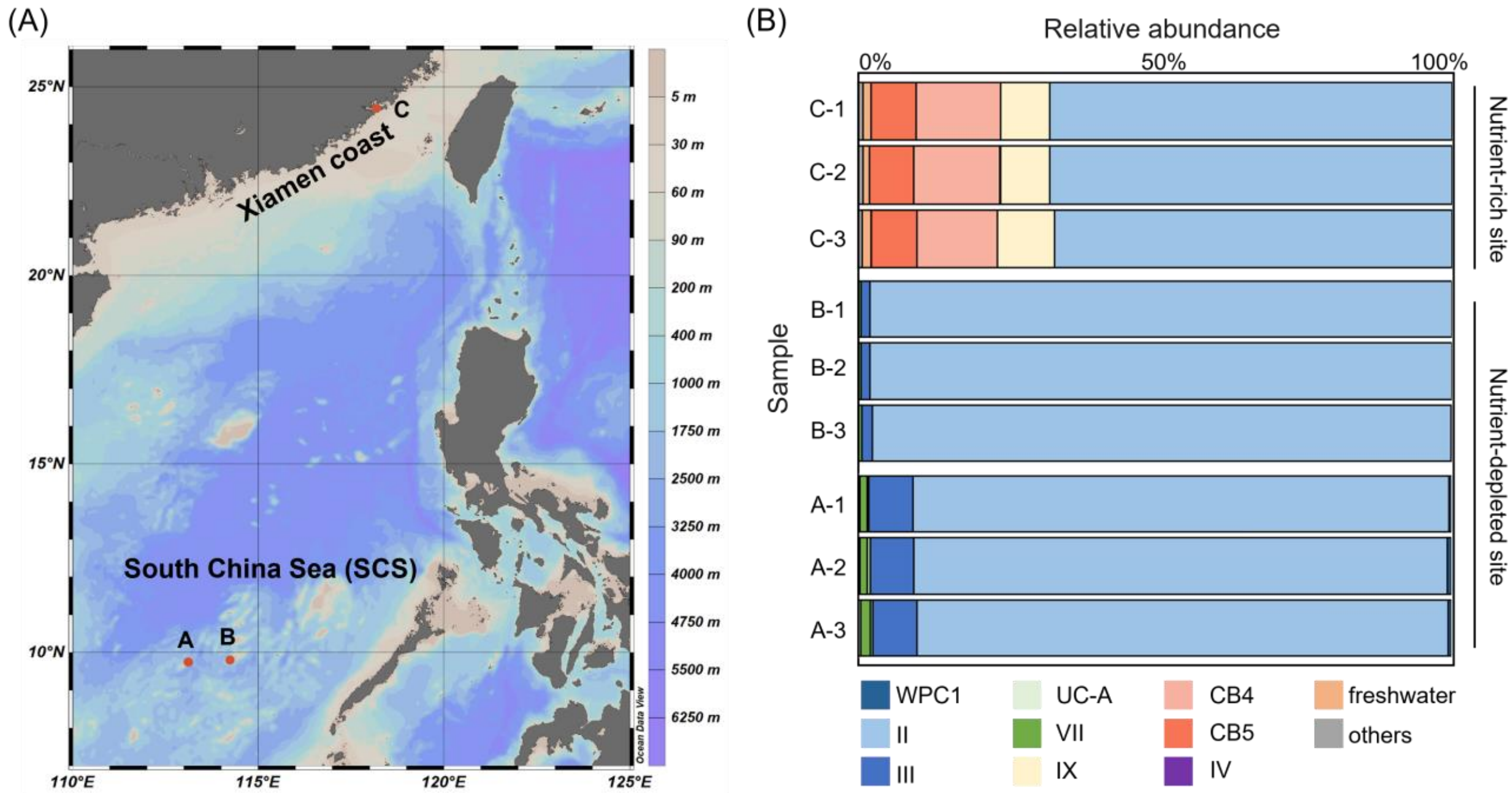

Fig. S1. Sample location and *Synechococcus* population composition. (A) Locations of study stations in the South China Sea and Xiamen coast. **(B)** Relative abundance of different *Synechococcus* clades across sampling sites (n = 3).

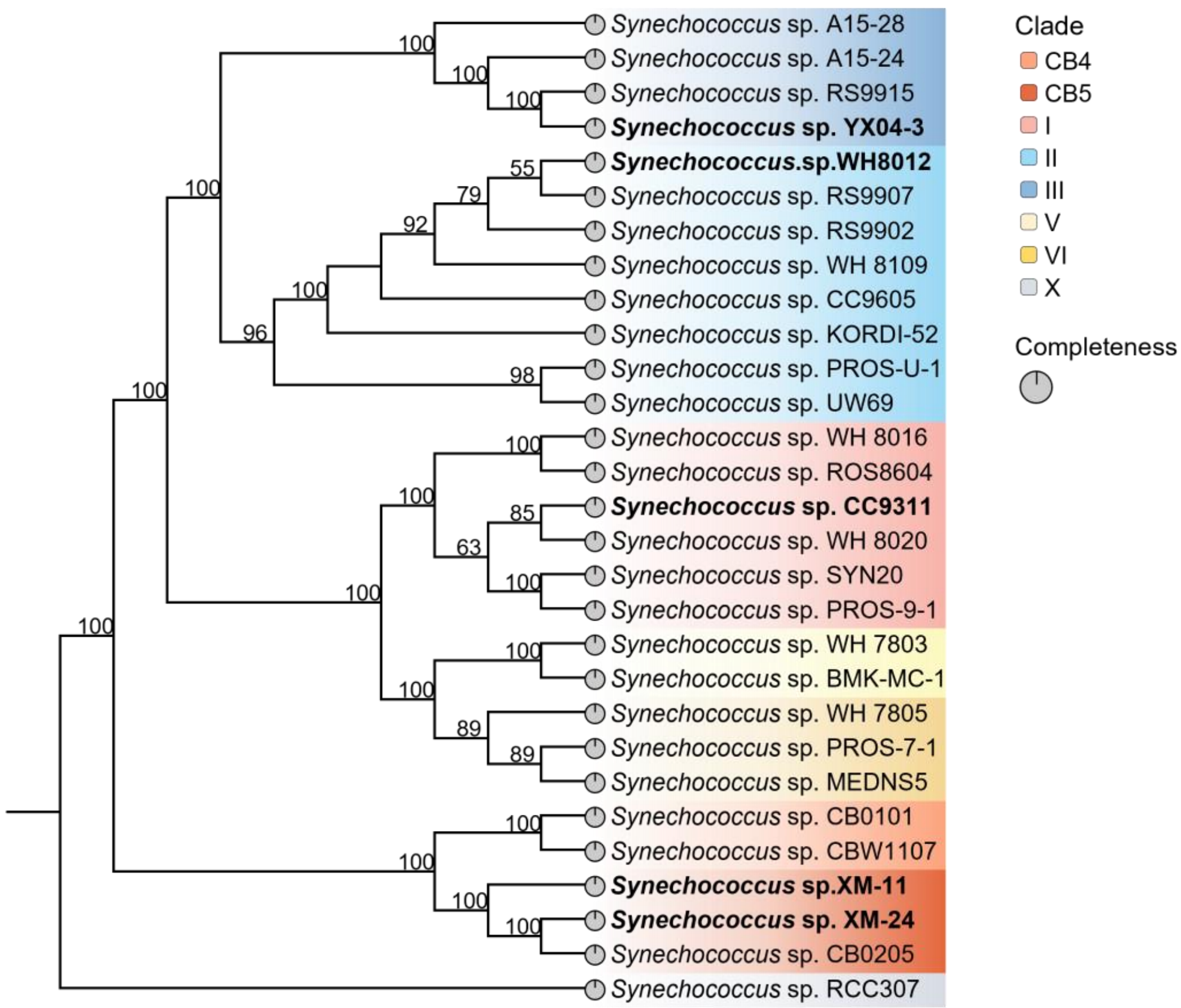

Fig. S2. Phylogenetic tree of selected *Synechococcus* strains. Phylogenetic tree was generated using 29 nearly complete genome sequences of *Synechococcus* from nutrient-depleted-adapted (12 genomes, II and III) and nutrient-rich-adapted (16 genomes, I, V, VI, CB4 and CB5) clades. Pie charts show the completeness of genomes. Bold strain names highlight the five strains used in laboratory experiments.

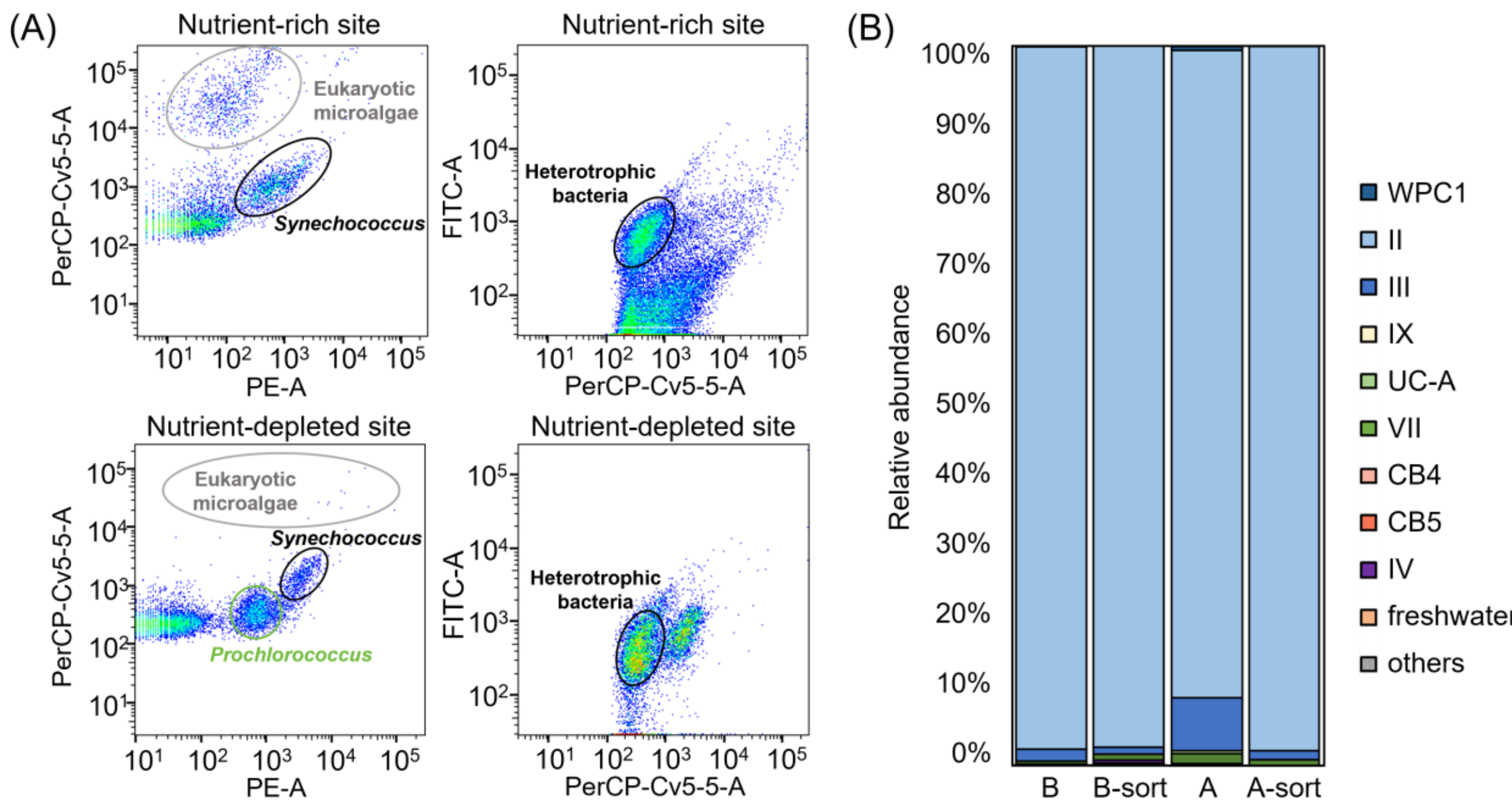

Fig. S3. Flow cytometry sorting accuracy. (A) Flow cytometry sorting plots with gates for different populations. **(B)** Comparison of *in situ* (n = 3) and sorted *Synechococcus* clade composition in our nutrient-depleted stations**.** A-sort and B-sort represent sorted *Synechococcus* cells from A and B stations.

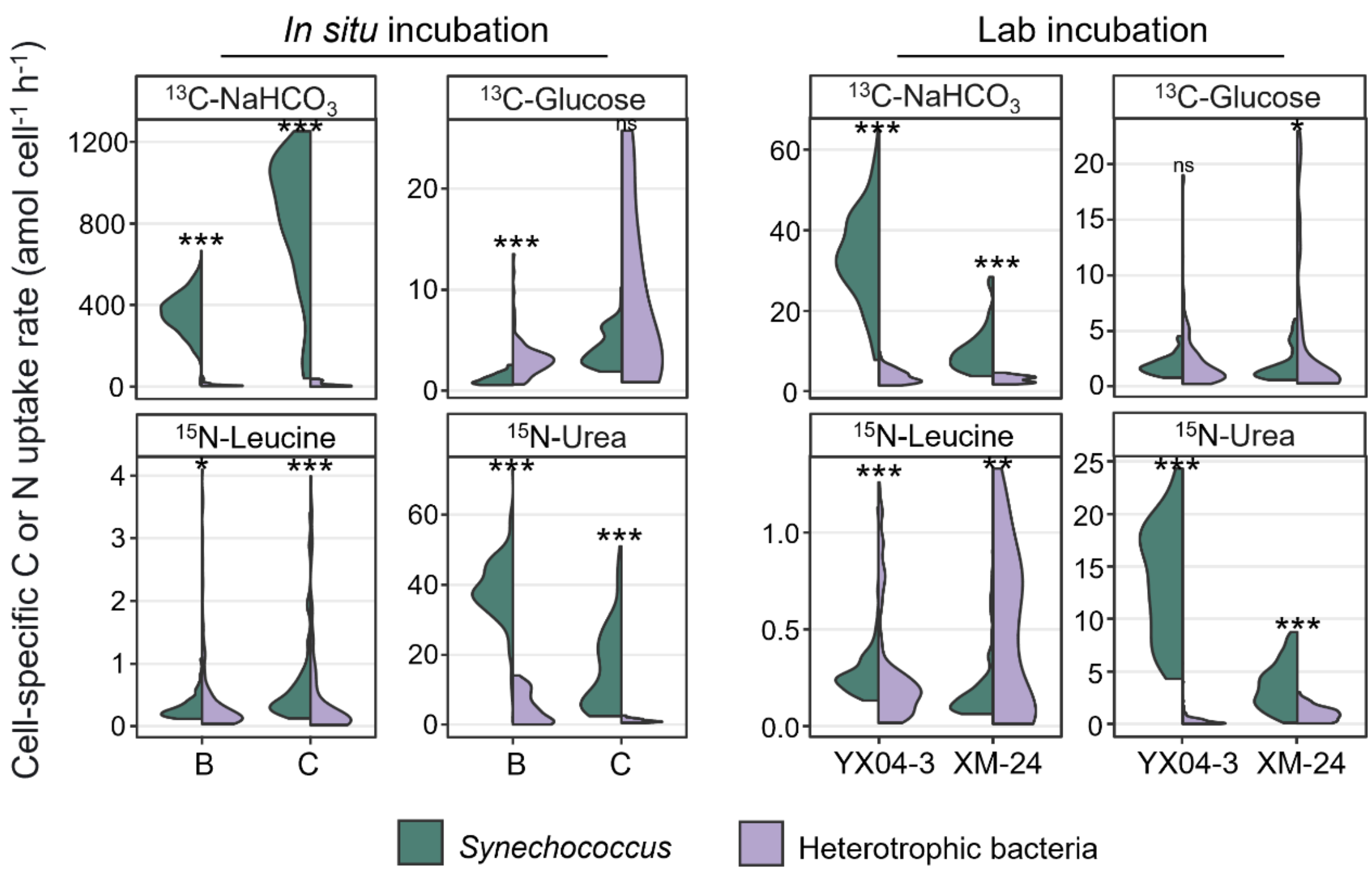

Fig. S4. Single-cell C- or N-specific C-fixation, glucose, leucine and urea uptake rates (amol cell^{-1} h^{-1}) in *in situ* and laboratory-cultured *Synechococcus* and co-existing heterotrophic bacteria. Station B (nutrient-depleted site), Station C (nutrient-rich site), *Synechococcus* sp. YX04-3 (nutrient-depleted strain, clade III), and *Synechococcus* sp. XM-24 (nutrient-rich strain, clade CB5) were selected as representative stations or laboratory cultures for comparative analysis. Asterisks denote statistical significance between *Synechococcus* (green) and heterotrophic bacteria (purple) uptake rates ($p < 0.05$ *, $p < 0.01$ **, $p < 0.001$ ***). For all statistical comparisons, exact *p*-values and *n* values are provided in Table S13.

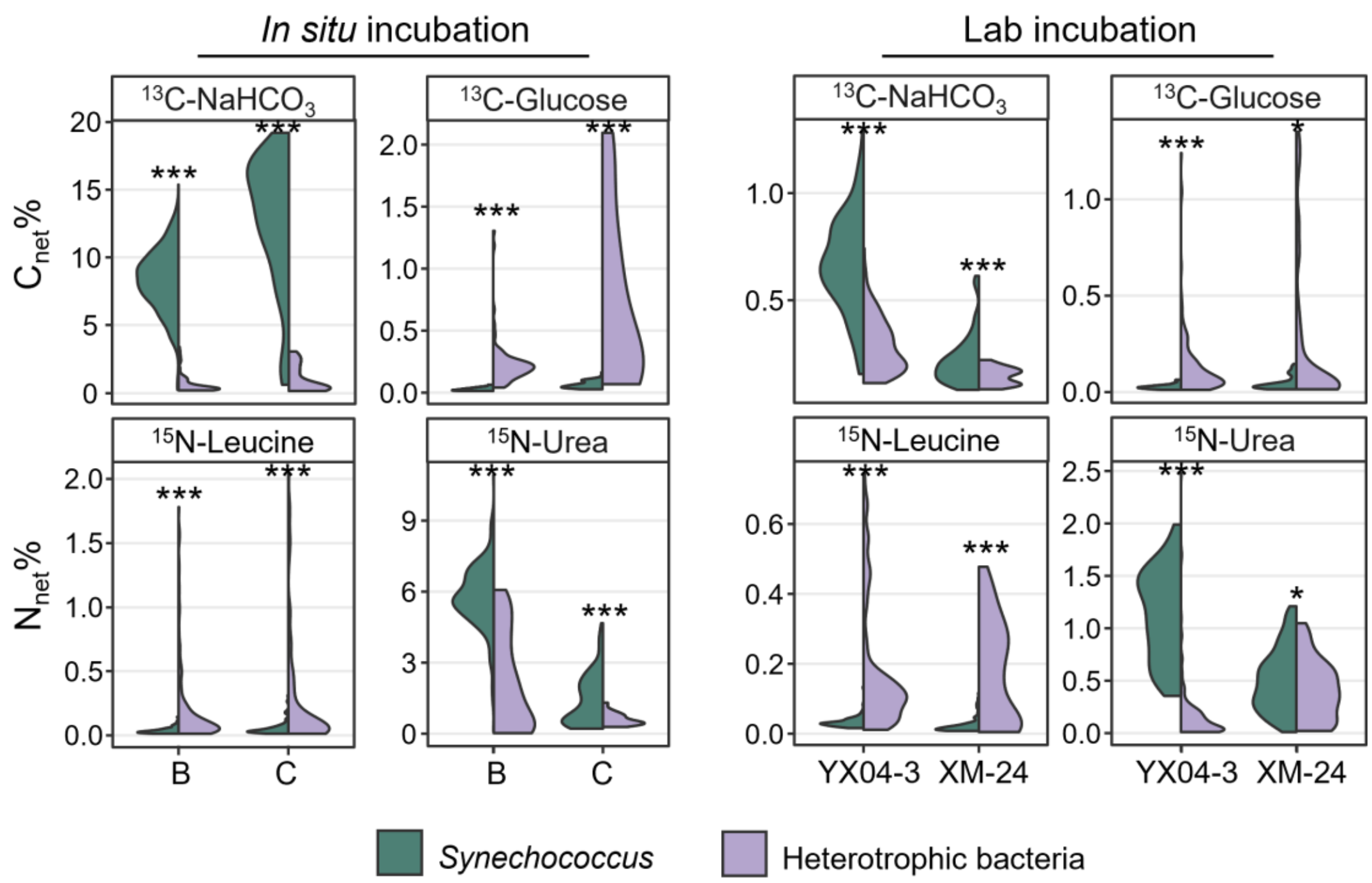

Fig. S5. Cell-specific net C or N assimilation percentages (C_{net}% or N_{net}%) for C-fixation, glucose, leucine and urea in *in situ* and laboratory-cultured *Synechococcus* and co-existing heterotrophic bacteria. Station B (nutrient-depleted site), Station C (nutrient-rich site), *Synechococcus* sp. YX04-3 (nutrient-depleted strain, clade III), and *Synechococcus* sp. XM-24 (nutrient-rich strain, clade CB5) were selected as representative stations or laboratory cultures for comparative analysis. Asterisks denote statistical significance between *Synechococcus* (green) and heterotrophic bacteria (purple) X_{net}% ($p < 0.05$ *, $p < 0.01$ **, $p < 0.001$ ***). For all statistical comparisons, exact *p*-values and *n* values are provided in Table S13.

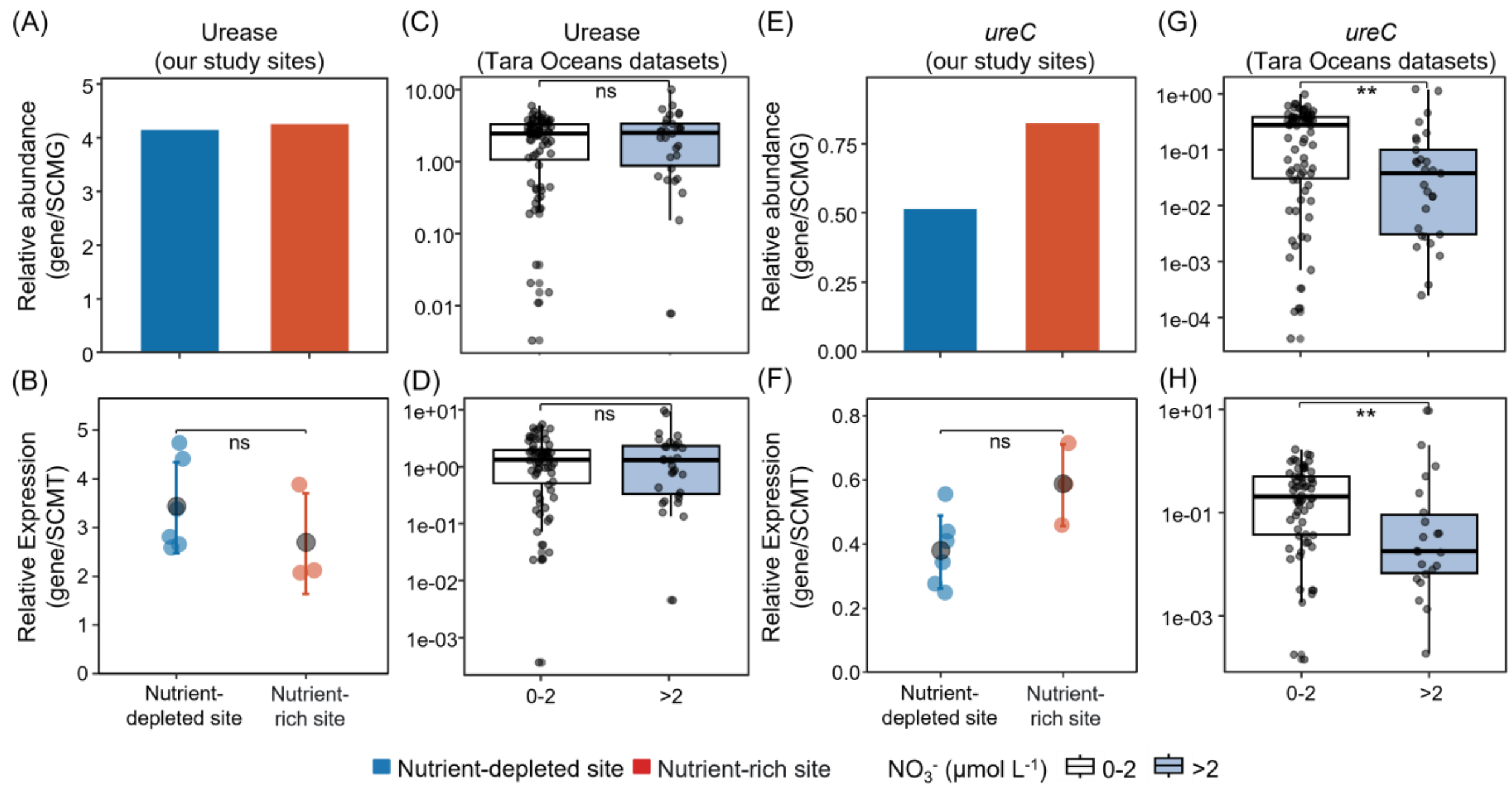

Fig. S6. Relative abundance and expression of *ureC* gene and urease gene operon in *Synechococcus* from study sites and Tara Oceans datasets. (A, B) Relative abundance and expression of urease (*ureABCDEFG*) between nutrient-depleted (blue, n = 6, metatranscriptomic data) and nutrient-rich (red, n = 3, metatranscriptomic data) sites. **(C, D)** Relative abundance and expression of the urease catalytic subunit gene *ureC* between nutrient-depleted (n = 6) and nutrient-rich (n = 3) sites. Black dots denote mean values of relative expression, with error bars showing standard deviation (SD). **(E, F)** Boxplots showing relative abundance (n = low-N: 60; high-N: 30) and expression (n = 57; 30) of urease between low-N (NO_3^- = 0–2 μmol L^{-1}) and high-N ($NO_3^- >$ 2 μmol L^{-1}) Tara Oceans regions. **(G, H)** Boxplots showing relative abundance (n = 59; 27) and expression (n = 55; 20) of *ureC* between low-N and high-N regions. Asterisks denote statistical significance ($p < 0.05$ *, $p < 0.01$ **, $p < 0.001$ ***).

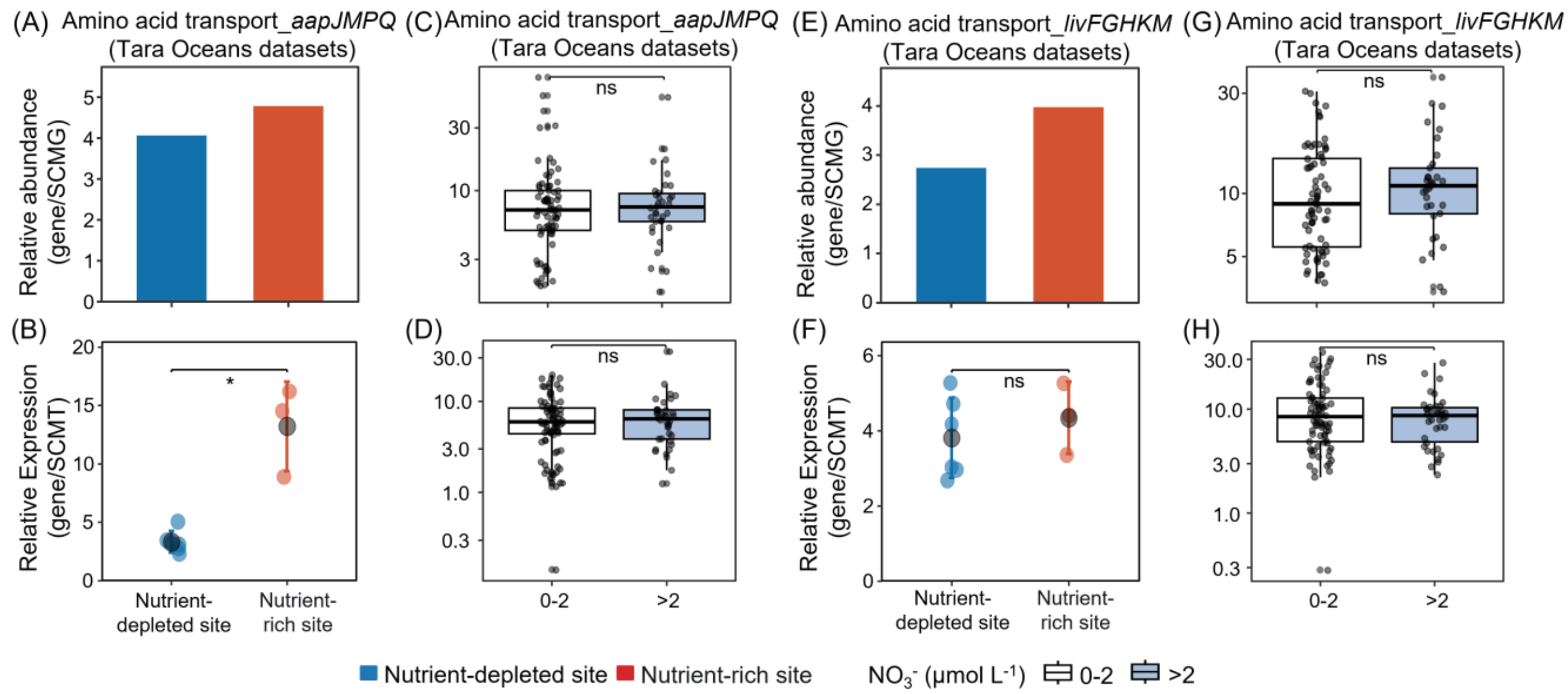

Fig. S7. Relative abundance and expression of amino acid transporters (*aapJMPQ* and *livFGHKM*) in *Synechococcus* from Tara Oceans datasets. (A, B) Relative abundance and expression of *aapJMPQ* between nutrient-depleted (blue, *n* = 6, metatranscriptomic data) and nutrient-rich (red, *n* = 3, metatranscriptomic data) sites. **(C, D)** Relative abundance and expression of *livFGHKM* between nutrient-depleted (*n* = 6) and nutrient-rich (*n* = 3) sites. Black dots denote mean values of relative expression, with error bars showing standard deviation (SD). **(E, F)** Boxplots showing relative abundance (*n* = low-N: 63; high-N: 34) and expression (*n* = 74; 38) of *aapJMPQ* between low-N (NO_3^- = 0–2 μmol L^{-1}) and high-N (NO_3^- > 2 μmol L^{-1}) Tara Oceans regions. **(G, H)** Boxplots showing relative abundance (*n* = 63; 34) and expression (*n* = 74; 38) of *livFGHKM* between low-N and high-N regions. Asterisks denote statistical significance ($p < 0.05$ *, $p < 0.01$ **, $p < 0.001$ ***).

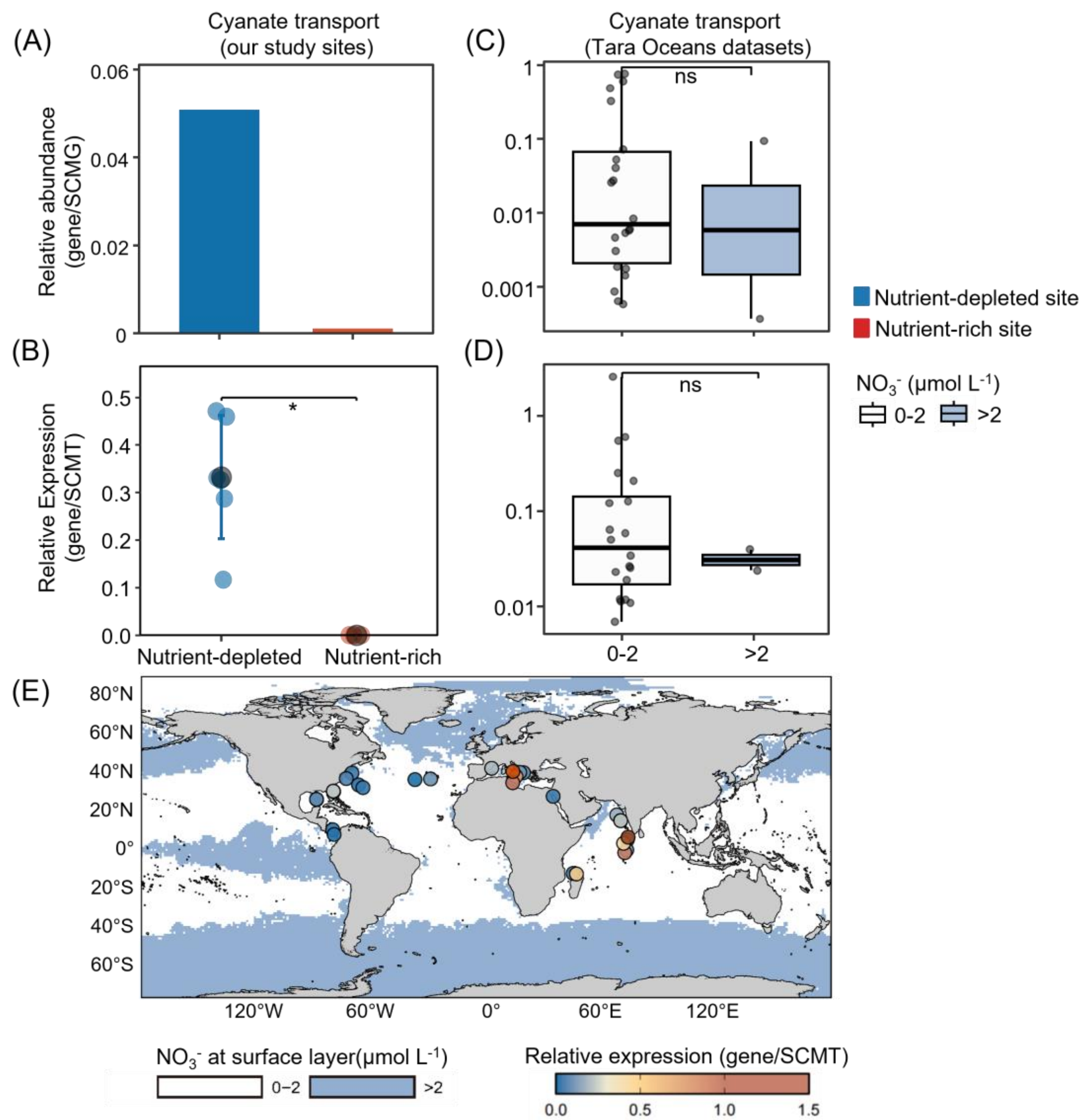

Fig. S8. Relative abundance and expression of the cyanate transporter (*cynABD*) in *Synechococcus* from our study sites and Tara Oceans datasets. (A, B) Relative abundance and expression of the cyanate transporter between nutrient-depleted (blue, n = 6, metatranscriptomic data) and nutrient-rich (red, n = 3, metatranscriptomic data) sites. Black dots denote mean values of relative expression, with error bars showing standard deviation (SD). **(C, D)** Boxplots of relative

abundance (n = low-N: 22; high-N: 2) and expression (n = 20; 2) of *cynABD* between low-N (NO_3^- = 0–2 µmol L^{-1}) and high-N ($NO_3^- > 2$ µmol L^{-1}) Tara Oceans regions. **(E)** Global NO_3^- distribution and relative expression of the cyanate transporter across Tara Oceans stations (n = 22). Asterisks denote statistical significance ($p < 0.05$ *, $p < 0.01$ **, $p < 0.001$ ***).

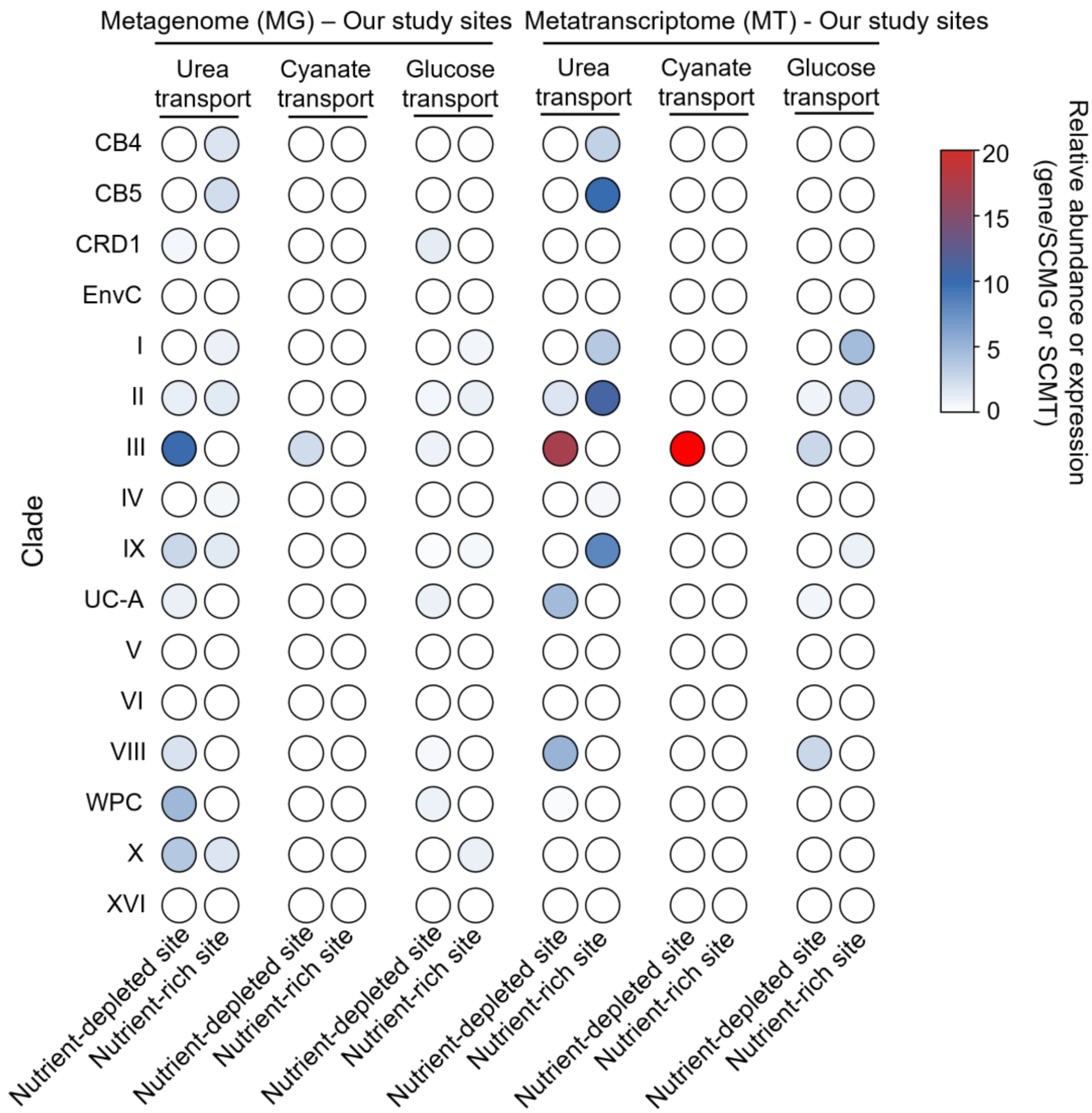

Fig. S9. Heatmap shows relative abundance and expression (*n* = 3) of urea (*urtABCDE* and *DUR3*), cyanate (*cynABD*) and glucose (*glcH*, *glcP*, and TCDB transporters) transporter genes in different *Synechococcus* clades from our nutrient-depleted and nutrient-rich study sites.

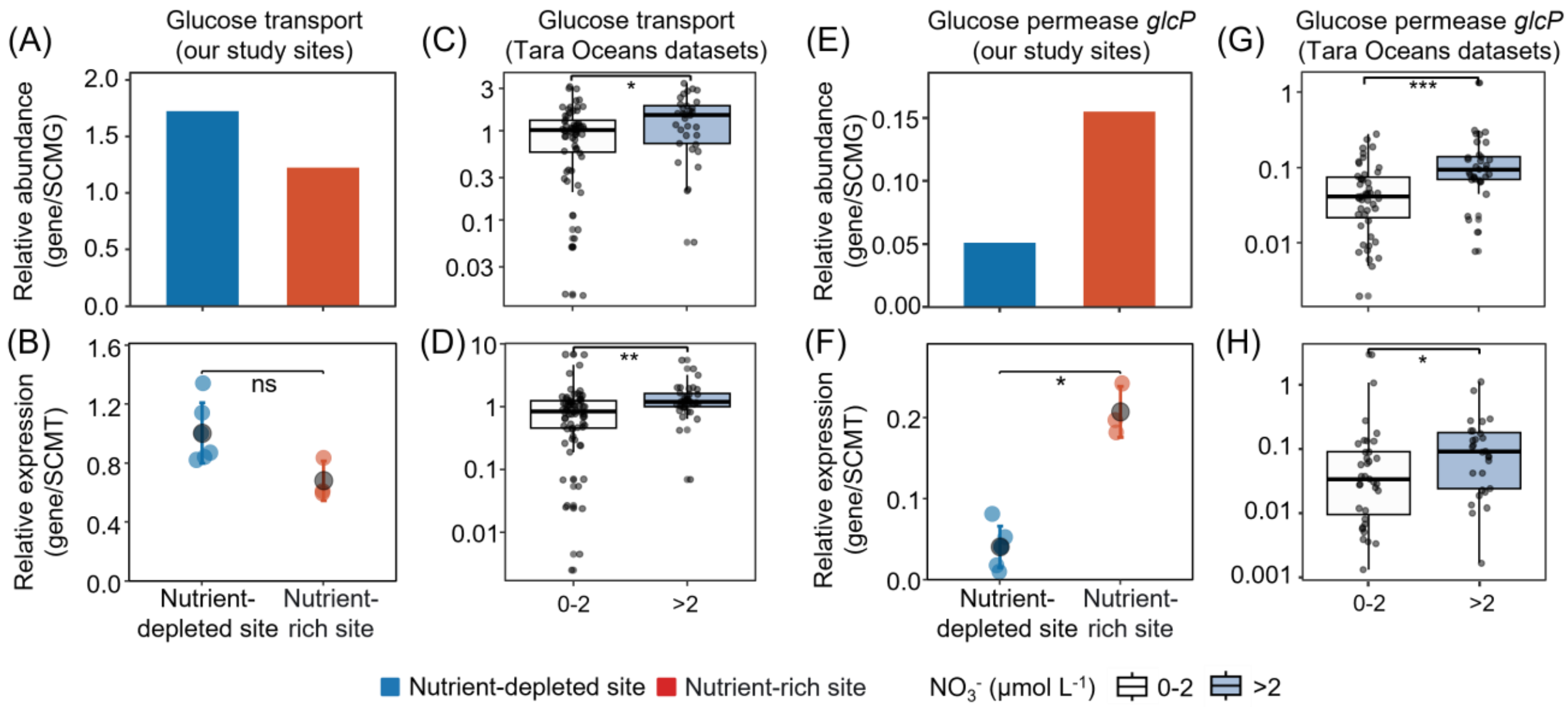

Fig. S10. Relative abundance and expression of glucose transporters in *Synechococcus* from our study sites and Tara Oceans datasets. (A, B) Relative abundance and expression of total glucose transporters (*glcH*, *glcP*, and TCDB glucose transporters) between nutrient-depleted (blue, $n = 6$, metatranscriptomic data) and nutrient-rich (red, $n = 3$, metatranscriptomic data) sites. **(C, D)** Relative abundance and expression of glucose permease *glcP* between nutrient-depleted ($n = 6$, metatranscriptomic data) and nutrient-rich ($n = 3$, metatranscriptomic data) sites. Black dots denote mean values of relative expression, with error bars showing standard deviation (SD). **(E, F)** Boxplots showing relative abundance (n = low-N: 63; high-N: 34) and expression (n = 71; 36) of total glucose transporters between low-N ($NO_3^- = 0$–2 μmol L^{-1}) and high-N ($NO_3^- > 2$ μmol L^{-1}) Tara Oceans regions. **(G, H)** Boxplots showing relative abundance (n = 48; 33) and expression (n = 36; 28) of *glcP* between low-N and high-N regions. Asterisks denote statistical significance ($p < 0.05$ *, $p < 0.01$ **, $p < 0.001$ ***).

Table S1. (separate Excel file)

Biogeochemical parameters of sampling stations and lab cultures (mean value of five studied *Synechococcus* strains).

Table S2. (separate Excel file)

Biochemical parameters and coefficients of variation (CV) of triplicate samples from each station.

Table S3. (separate Excel file)

The final concentration of added isotope-labeled substrates and correction factors of glucose and urea uptake rates for field incubations and laboratory cultures.

Table S4. (separate Excel file)

Summary of C:N ratio of *Synechococcus* from previous studies.

Table S5. (separate Excel file)

Information of 669 qualified genomes.

Table S6. (separate Excel file)

Genes involved in different substrate transport or utilization.

Table S7. (separate Excel file)

Single-cell C-fixation, glucose, leucine, and urea uptake rates (amol $cell^{-1}$ h^{-1}) in *in situ* and laboratory-cultured *Synechococcus*, summarized by nutrient-depleted and nutrient-rich sites and

strains. Data are shown as median (Q1, Q3). Data in brackets: [active count/total count, percentage of active cells].

Table S8. (separate Excel file)

Cell-specific net C or N assimilation percentages (C_{net}% or N_{net}%) in *in situ* and laboratory-cultured *Synechococcus*, summarized by nutrient-depleted and nutrient-rich sites and strains. Data are shown as median (Q1, Q3).

Table S9. (separate Excel file)

Single-cell C-fixation, glucose, leucine, and urea uptake rates (amol $cell^{-1}$ h^{-1}) in *Synechococcus* (SYN) and heterotrophic bacteria (HB) from individual stations and laboratory strains. HB data are provided only for stations and strains included in paired SYN-HB experiments. Data are shown as median (Q1, Q3). Data in brackets: [active count/total count, percentage of active cells].

Table S10. (separate Excel file)

Cell-specific net C or N assimilation percentages (C_{net}% or N_{net}%) in *Synechococcus* (SYN) and heterotrophic bacteria (HB) from individual stations and laboratory strains. HB data are provided only for stations and strains included in paired SYN-HB experiments. Data are shown as median (Q1, Q3).

Table S11. (separate Excel file)

Relative abundance of specific transporters and their contribution to total nitrogen transport in our

study sites and Tara Oceans datasets (depth in SRF and DCM).

Table S12. (separate Excel file)

Relative expression of specific transporters and their contribution to total nitrogen transport in our study sites and Tara Oceans datasets (depth in SRF and DCM).

Table S13. (separate Excel file)

Statistical summary of Wilcoxon rank-sum tests for cell-specific nutrient uptake rates and X_{net}%. Comparisons are provided between (i) nutrient-depleted and nutrient-rich sites, (ii) nutrient-depleted and nutrient-rich *Synechococcus* strains, and (iii) *Synechococcus* and heterotrophic bacteria. Exact *p* values and sample sizes (*n*) are reported for all comparisons.

Data S1. (separate ZIP file)

HMM files used for single-copy gene, *glcP* and N-related gene identification.

REFERENCES

1. P. Flombaum, J. L. Gallegos, R. A. Gordillo, J. Rincón, L. L. Zabala, N. Jiao, D. M. Karl, W. K. W. Li, M. W. Lomas, D. Veneziano, C. S. Vera, J. A. Vrugt, A. C. Martiny, Present and future global distributions of the marine Cyanobacteria *Prochlorococcus* and *Synechococcus*. *Proc. Natl. Acad. Sci. U.S.A.* **110**, 9824–9829 (2013).

2. K. Schmidt, A. J. Birchill, A. Atkinson, R. J. W. Brewin, J. R. Clark, A. E. Hickman, D. G. Johns, M. C. Lohan, A. Milne, S. Pardo, L. Polimene, T. J. Smyth, G. A. Tarran, C. E. Widdicombe, E. M. S. Woodward, S. J. Ussher, Increasing picocyanobacteria success in shelf waters contributes to long-term food web degradation. *Glob. Change Biol.* **26**, 5574–5587 (2020).

3. F. P. Chavez, M. Messié, J. T. Pennington, Marine primary production in relation to climate variability and change. *Ann. Rev. Mar. Sci.* **3**, 227–260 (2011).

4. D. J. Scanlan, M. Ostrowski, S. Mazard, A. Dufresne, L. Garczarek, W. R. Hess, A. F. Post, M. Hagemann, I. Paulsen, F. Partensky, Ecological genomics of marine picocyanobacteria. *Microbiol. Mol. Biol. Rev.* **73**, 249–299 (2009).

5. N. A. Kamennaya, A. F. Post, Characterization of cyanate metabolism in marine *Synechococcus* and *Prochlorococcus* spp. *Appl. Environ. Microbiol.* **77**, 291–301 (2011).

6. M. C. Muñoz-Marín, G. Gómez-Baena, A. López-Lozano, J. A. Moreno-Cabezuelo, J. Díez, J. M. García-Fernández, Mixotrophy in marine picocyanobacteria: Use of organic compounds by *Prochlorococcus* and *Synechococcus*. *ISME J.* **14**, 1065–1073 (2020).

7. S. Duhamel, F. Van Wambeke, D. Lefevre, M. Benavides, S. Bonnet, Mixotrophic metabolism by natural communities of unicellular cyanobacteria in the western tropical South Pacific Ocean. *Environ. Microbiol.* **20**, 2743–2756 (2018).

8. H. Berthelot, S. Duhamel, S. L'Helguen, J. Maguer, N. Cassar, Inorganic and organic carbon and nitrogen uptake strategies of picoplankton groups in the northwestern Atlantic Ocean. *Limnol. Oceanogr.* **66**, 3682–3696 (2021).

9. H. Berthelot, S. Duhamel, S. L'Helguen, J.-F. Maguer, S. Wang, I. Cetinić, N. Cassar, NanoSIMS single cell analyses reveal the contrasting nitrogen sources for small phytoplankton. *ISME J.* **13**, 651–662 (2018).

10. I. N. Shilova, M. M. Mills, J. C. Robidart, K. A. Turk-Kubo, K. M. Björkman, Z. Kolber, I. Rapp, G. L. van Dijken, M. J. Church, K. R. Arrigo, E. P. Achterberg, J. P. Zehr, Differential effects of nitrate, ammonium, and urea as N sources for microbial communities in the north Pacific Ocean. *Limnol. Oceanogr.* **62**, 2550–2574 (2017).

11. L. R. Moore, More mixotrophy in the marine microbial mix. *Proc. Natl. Acad. Sci. U.S.A.* **110**, 8323–8324 (2013).

12. M. D. C. Muñoz-Marín, I. Luque, M. V. Zubkov, P. G. Hill, J. Diez, J. M. García-Fernández, *Prochlorococcus* can use the Pro1404 transporter to take up glucose at nanomolar concentrations in the Atlantic Ocean. *Proc. Natl. Acad. Sci. U.S.A.* **110**, 8597–8602 (2013).

13. D. E. Michels, B. Lomenick, T.-F. Chou, M. J. Sweredoski, A. Pasulka, Amino acid analog induces stress response in marine *Synechococcus*. *Appl. Environ. Microbiol.* **87**, e0020021 (2021).

14. M. Aldunate, C. Henríquez-Castillo, Q. Ji, J. Lueders-Dumont, M. R. Mulholland, B. B. Ward, P. von Dassow, O. Ulloa, Nitrogen assimilation in picocyanobacteria inhabiting the oxygen-deficient waters of the eastern tropical north and south Pacific. *Limnol. Oceanogr.* **65**, 437–453 (2020).

15. J. A. Sohm, N. A. Ahlgren, Z. J. Thomson, C. Williams, J. W. Moffett, M. A. Saito, E. A. Webb, G. Rocap, Co-occurring *Synechococcus* ecotypes occupy four major oceanic regimes defined by temperature, macronutrients and iron. *ISME J.* **10**, 333–345 (2016).

16. F. Chen, Picocyanobacteria in the Chesapeake Bay: Isolation, diversity, and adaptation. *Mar. Life Sci. Technol.* **7**, 434–449 (2025).

17. C. Wang, W. Zhu, H. Ding, N. Liu, H. Cao, C. Suo, Z. Liu, Y. Zhang, M. Sun, H. Fu, C. Li, X. Chen, Y.-Z. Zhang, P. Wang, Structural and molecular basis for urea recognition by *Prochlorococcus*. *J. Biol. Chem.* **299**, 104958 (2023).

18. D. J. Scanlan, Physiological diversity and niche adaptation in marine *Synechococcus*. *Adv. Microb. Physiol.* **47**, 1–64 (2003).

19. T. D. Jickells, Nutrient biogeochemistry of the coastal zone. *Science* **281**, 217–222 (1998).

20. J. Carstensen, R. Klais, J. E. Cloern, Phytoplankton blooms in estuarine and coastal waters: Seasonal patterns and key species. *Estuar. Coast. Shelf Sci.* **162**, 98–109 (2015).

21. B. Palenik, Q. Ren, C. L. Dupont, G. S. Myers, J. F. Heidelberg, J. H. Badger, R. Madupu, W. C. Nelson, L. M. Brinkac, R. J. Dodson, A. S. Durkin, S. C. Daugherty, S. A. Sullivan, H. Khouri, Y. Mohamoud, R. Halpin, I. T. Paulsen, Genome sequence of *Synechococcus* CC9311: Insights into adaptation to a coastal environment. *Proc. Natl. Acad. Sci. U.S.A.* **103**, 13555–13559 (2006).

22. T. Berman, D. A. Bronk, Dissolved organic nitrogen: A dynamic participant in aquatic ecosystems. *Aquat. Microb. Ecol.* **31**, 279–305 (2003).

23. Z. Wu, D. Aharonovich, D. Roth-Rosenberg, O. Weissberg, T. Luzzatto-Knaan, A. Vogts, L. Zoccarato, F. Eigemann, H.-P. Grossart, M. Voss, M. J. Follows, D. Sher, Single-cell measurements and modelling reveal substantial organic carbon acquisition by *Prochlorococcus*. *Nat. Microbiol.* **7**, 2068–2077 (2022).

24. Q. Zheng, W. Lin, Y. Wang, Y. Li, C. He, Y. Shen, W. Guo, Q. Shi, N. Jiao, Highly enriched N-containing organic molecules of *Synechococcus* lysates and their rapid transformation by heterotrophic bacteria. *Limnol. Oceanogr.* **66**, 335–348 (2021).

25. C. Solomon, J. Collier, G. Berg, P. Glibert, Role of urea in microbial metabolism in aquatic systems: A biochemical and molecular review. *Aquat. Microb. Ecol.* **59**, 67–88 (2010).

26. A. E. Zimmerman, J. C. Podowski, G. E. Gallagher, M. L. Coleman, J. R. Waldbauer, Tracking nitrogen allocation to proteome biosynthesis in a marine microbial community. *Nat. Microbiol.* **8**, 498–509 (2023).

27. M. D. C. Muñoz-Marín, A. López-Lozano, J. Á. Moreno-Cabezuelo, J. Díez, J. M. García-Fernández, Mixotrophy in cyanobacteria. *Curr. Opin. Microbiol.* **78**, 102432 (2024).

28. E. A. Groisman, Feedback control of two-component regulatory systems. *Annu. Rev. Microbiol.* **70**, 103–124 (2016).

29. D. K. Button, Nutrient-limited microbial growth kinetics: Overview and recent advances. *Antonie Van Leeuwenhoek* **63**, 225–235 (1993).

30. M. D. C. Muñoz-Marín, S. Duhamel, K. M. Björkman, J. D. Magasin, J. Díez, D. M. Karl, J. M. García-Fernández, Differential timing for glucose assimilation in *Prochlorococcus* and coexistent microbial populations in the north Pacific subtropical gyre. *Microbiol. Spectr.* **10**, e0246622 (2022).

31. J. Á. Moreno-Cabezuelo, G. Gómez-Baena, J. Díez, J. M. García-Fernández, Integrated proteomic and metabolomic analyses show differential effects of glucose availability in marine *Synechococcus* and *Prochlorococcus*. *Microbiol. Spectr.* **11**, e03275–22 (2023).

32. H. Doré, U. Guyet, J. Leconte, G. K. Farrant, B. Alric, M. Ratin, M. Ostrowski, M. Ferrieux, L. Brillet-Guéguen, M. Hoebeke, J. Siltanen, G. Le Corguillé, E. Corre, P. Wincker, D. J. Scanlan, D. Eveillard, F. Partensky, L. Garczarek, Differential global distribution of marine picocyanobacteria gene clusters reveals distinct niche-related adaptive strategies. *ISME J.* **17**, 720–732 (2023).

33. B. Xue, P. Sartori, S. Leibler, Environment-to-phenotype mapping and adaptation strategies in varying environments. *Proc. Natl. Acad. Sci. U.S.A.* **116**, 13847–13855 (2019).

34. Q. Zheng, Y. Wang, J. Lu, W. Lin, F. Chen, N. Jiao, Metagenomic and metaproteomic insights into photoautotrophic and heterotrophic interactions in a *Synechococcus* culture. *MBio* **11**, e03261–19 (2020).

35. N. Kamjunke, B. Köhler, N. Wannicke, J. Tittel, Algae as competitors for glucose with heterotrophic bacteria. *J. Phycol.* **44**, 616–623 (2008).

36. W. Deng, S. Wang, X. Wan, Z. Zheng, N. Jiao, S.-J. Kao, J. K. Moore, Y. Zhang, Potential competition between marine heterotrophic prokaryotes and autotrophic picoplankton for nitrogen substrates. *Limnol. Oceanogr.* **66**, 3338–3355 (2021).

37. G. Fusco, A. Minelli, Phenotypic plasticity in development and evolution: Facts and concepts. *Philos. Trans. R. Soc. B Biol. Sci.* **365**, 547–556 (2010).

38. J. J. McCarthy, W. R. Taylor, J. L. Taft, Nitrogenous nutrition of the plankton in the Chesapeake Bay. 1. Nutrient availability and phytoplankton preferences. *Limnol. Oceanogr.* **22**, 996–1011 (1977).

39. N. A. Kamennaya, M. Chernihovsky, A. F. Post, The cyanate utilization capacity of marine unicellular Cyanobacteria. *Limnol. Oceanogr.* **53**, 2485–2494 (2008).

40. J. J. Polovina, E. A. Howell, M. Abecassis, Ocean's least productive waters are expanding. *Geophys. Res. Lett.* **35**, L03618 (2008).

41. A. Tsiola, P. Pitta, S. Fodelianakis, R. Pete, I. Magiopoulos, P. Mara, S. Psarra, T. Tanaka, B. Mostajir, Nutrient limitation in surface waters of the oligotrophic eastern Mediterranean Sea: An enrichment microcosm experiment. *Microb. Ecol.* **71**, 575–588 (2016).

42. E. Druce, S. C. Maberly, P. Sánchez-Baracaldo, Wide-ranging organic nitrogen diets of freshwater picocyanobacteria. *ISME J.* **19**, wrae236 (2025).

43. F. M. M. Morel, Kinetics of nutrient uptake and growth in phytoplankton. *J. Phycol.* **23**, 137–150 (1987).

44. B. E. Clifton, U. Alcolombri, G.-I. Uechi, C. J. Jackson, P. Laurino, The ultra-high affinity transport proteins of ubiquitous marine bacteria. *Nature* **634**, 721–728 (2024).

45. D. A. Bronk, J. H. See, P. Bradley, L. Killberg, DON as a source of bioavailable nitrogen for phytoplankton. *Biogeosciences* **4**, 283–296 (2007).

46. J. M. Wood, Leucine transport in *Escherichia coli*. The resolution of multiple transport systems and their coupling to metabolic energy. *J. Biol. Chem.* **250**, 4477–4485 (1975).

47. M. D. C. Muñoz-Marín, G. Gómez-Baena, J. Díez, R. J. Beynon, D. González-Ballester, M. V. Zubkov, J. M. García-Fernández, Glucose uptake in *Prochlorococcus*: Diversity of kinetics and effects on the metabolism. *Front. Microbiol.* **8**, 327 (2017).

48. S. Picossi, E. Flores, M. Ekman, Diverse roles of the GlcP glucose permease in free-living and symbiotic Cyanobacteria. *Plant Signal. Behav.* **8**, e27416 (2013).

49. X. Mao, J. Chen, C. van Oosterhout, H. Zhang, G. Liu, Y. Zhuang, T. Mock, Diversity, prevalence, and expression of cyanase genes (*cynS*) in planktonic marine microorganisms. *ISME J.* **16**, 602–605 (2022).

50. G. Capovilla, R. Braakman, G. P. Fournier, T. Hackl, J. Schwartzman, X. Lu, A. Yelton, K. Longnecker, M. C. K. Soule, E. Thomas, G. Swarr, A. Mongera, J. G. Payette, K. G. Castro, J. R. Waldbauer, E. B. Kujawinski, O. X. Cordero, S. W. Chisholm, Chitin utilization by marine picocyanobacteria and the evolution of a planktonic lifestyle. *Proc. Natl. Acad. Sci. U.S.A.* **120**, e2213271120 (2023).

51. S.-Q. Li, H.-L. Huang, T.-R. Sun, H.-Y. Gao, X.-W. Wang, F.-X. Fu, D. A. Hutchins, H.-B. Jiang, Cyanobacteria using urea as a nitrogen source can overcome acid stress. bioRxiv 2023.03.29.534730 [Preprint] (2023). https://doi.org/10.1101/2023.03.29.534730.

52. Y.-Y. Li, X.-H. Chen, C. Xue, H. Zhang, G. Sun, Z.-X. Xie, L. Lin, D.-Z. Wang, Proteomic response to rising temperature in the marine cyanobacterium *Synechococcus* grown in different nitrogen sources. *Front. Microbiol.* **10**, 1976 (2019).

53. N. S. Fisher, R. A. Cowdell, Growth of marine planktonic diatoms on inorganic and organic nitrogen. *Mar. Biol.* **72**, 147–155 (1982).

54. X. Jing, S. Lin, H. Zhang, C. Koerting, Z. Yu, Utilization of urea and expression profiles of related genes in the dinoflagellate *Prorocentrum donghaiense*. *PLOS ONE* **12**, e0187837 (2017).

55. N. Visintini, A. C. Martiny, P. Flombaum, *Prochlorococcus*, *Synechococcus*, and picoeukaryotic phytoplankton abundances in the global ocean. *Limnol. Oceanogr. Lett.* **6**, 207–215 (2021).

56. R. W. Izett, K. Fennel, A. C. Stoer, D. P. Nicholson, Reviews and syntheses: Expanding the global coverage of gross primary production and net community production measurements using biogeochemical-argo floats. *Biogeosciences* **21**, 13–47 (2024).

57. C. M. Moore, M. M. Mills, K. R. Arrigo, I. Berman-Frank, L. Bopp, P. W. Boyd, E. D. Galbraith, R. J. Geider, C. Guieu, S. L. Jaccard, T. D. Jickells, J. La Roche, T. M. Lenton, N. M. Mahowald, E. Marañón, I. Marinov, J. K. Moore, T. Nakatsuka, A. Oschlies, M. A. Saito, T. F. Thingstad, A. Tsuda, O. Ulloa, Processes and patterns of oceanic nutrient limitation. *Nat. Geosci.* **6**, 701–710 (2013).

58. J. Stuehrenberg, K. Kitzinger, J. N. von Arx, J. S. Graf, G. Lavik, S. Littmann, J. Milucka, W. D. Orsi, S. Schorn, D. R. Speth, A. Vuillemin, S. Wu, H. K. Marchant, M. M. M. Kuypers, Urea use drives niche separation between dominant marine ammonia oxidizing archaea. *Nat. Commun.* **16**, 10946 (2025).

59. J. B. Waterbury, S. W. Watson, F. W. Valois, D. G. Franks, Biological and ecological characterization of the marine unicellular cyanobacterium *Synechococcus*, in *Photosynthetic Picoplankton*, vol. **214** of Canadian Bulletin of Fisheries and Aquatic Sciences, T. Platt, W. K. W. Li, Eds. (Department of Fisheries and Oceans, Ottawa, Canada, 1986), pp. 71–120.

60. J.-B. Raina, M. Giardina, D. R. Brumley, P. L. Clode, M. Pernice, P. Guagliardo, J. Bougoure, H. Mendis, S. Smriga, E. C. Sonnenschein, M. S. Ullrich, R. Stocker, J. R. Seymour, Chemotaxis increases metabolic exchanges between marine picophytoplankton and heterotrophic bacteria. *Nat. Microbiol.* **8**, 510–521 (2023).

61. G. Bratbak, I. Dundas, Bacterial dry matter content and biomass estimations. *Appl. Environ. Microbiol.* **48**, 755–757 (1984).

62. P. G. Verity, C. Y. Robertson, C. R. Tronzo, M. G. Andrews, J. R. Nelson, M. E. Sieracki, Relationships between cell volume and the carbon and nitrogen content of marine photosynthetic nanoplankton. *Limnol. Oceanogr.* **37**, 1434–1446 (1992).

63. N. Kroer, Relationships between biovolume and carbon and nitrogen content of bacterioplankton. *FEMS Microbiol. Ecol.* **13**, 217–223 (1994).

64. R. Popa, P. K. Weber, J. Pett-Ridge, J. A. Finzi, S. J. Fallon, I. D. Hutcheon, K. H. Nealson, D. G. Capone, Carbon and nitrogen fixation and metabolite exchange in and between individual cells of *Anabaena oscillarioides*. *ISME J.* **1**, 354–360 (2007).

65. A. E. Dekas, A. E. Parada, X. Mayali, J. A. Fuhrman, J. Wollard, P. K. Weber, J. Pett-Ridge, Characterizing chemoautotrophy and heterotrophy in marine archaea and bacteria with single-cell multi-isotope NanoSIP. *Front. Microbiol.* **10**, 698 (2019).

66. J. C. Goldman, J. J. McCarthy, D. G. Peavey, Growth rate influence on the chemical composition of phytoplankton in oceanic waters. *Nature* **279**, 210–215 (1979).

67. A. Le Bouteiller, Comparison of in-bottle measurements using 15N and 14C. *ICES Mar. Sci. Symp.* **197**, 121–131 (1993).

68. D. A. Hansell, C. A. Carlson, Net community production of dissolved organic carbon. *Global Biogeochem. Cycles* **12**, 443–453 (1998).

69. K. S. Johnson, M. R. Mazloff, M. B. Bif, Y. Takeshita, H. W. Jannasch, T. L. Maurer, J. N. Plant, A. Verdy, P. M. Walz, S. C. Riser, L. D. Talley, Carbon to nitrogen uptake ratios observed across the Southern Ocean by the SOCCOM profiling float array. *J. Geophys. Res. Oceans* **127**, e2022JC018859 (2022).

70. J. Chen, Y. Jia, Y. Sun, K. Liu, C. Zhou, C. Liu, D. Li, G. Liu, C. Zhang, T. Yang, L. Huang, Y. Zhuang, D. Wang, D. Xu, Q. Zhong, Y. Guo, A. Li, I. Seim, L. Jiang, L. Wang, S. M. Y. Lee, Y. Liu, D. Wang, G. Zhang, S. Liu, X. Wei, Z. Yue, S. Zheng, X. Shen, S. Wang, C. Qi, J. Chen, C. Ye, F. Zhao, J. Wang, J. Fan, B. Li, J. Sun, X. Jia, Z. Xia, H. Zhang, J. Liu, Y. Zheng, X. Liu, J.

Wang, H. Yang, K. Kristiansen, X. Xu, T. Mock, S. Li, W. Zhang, G. Fan, Global marine microbial diversity and its potential in bioprospecting. *Nature* **633**, 371–379 (2024).

71. D. H. Parks, M. Imelfort, C. T. Skennerton, P. Hugenholtz, G. W. Tyson, CheckM: Assessing the quality of microbial genomes recovered from isolates, single cells, and metagenomes. *Genome Res.* **25**, 1043–1055 (2015).

72. D. Hyatt, G.-L. Chen, P. F. LoCascio, M. L. Land, F. W. Larimer, L. J. Hauser, Prodigal: Prokaryotic gene recognition and translation initiation site identification. *BMC Bioinformatics* **11**, 119 (2010).

73. A. Milanese, D. R. Mende, L. Paoli, G. Salazar, H.-J. Ruscheweyh, M. Cuenca, P. Hingamp, R. Alves, P. I. Costea, L. P. Coelho, T. S. B. Schmidt, A. Almeida, A. L. Mitchell, R. D. Finn, J. Huerta-Cepas, P. Bork, G. Zeller, S. Sunagawa, Microbial abundance, activity and population genomic profiling with mOTUs2. *Nat. Commun.* **10**, 1014 (2019).

74. UniProt Consortium, UniProt: A worldwide hub of protein knowledge. *Nucleic Acids Res.* **47**, D506–D515 (2019).

75. M. H. Saier, V. S. Reddy, G. Moreno-Hagelsieb, K. J. Hendargo, Y. Zhang, V. Iddamsetty, K. J. K. Lam, N. Tian, S. Russum, J. Wang, A. Medrano-Soto, The transporter classification database (TCDB): 2021 update. *Nucleic Acids Res.* **49**, D461–D467 (2021).

76. L. Fu, B. Niu, Z. Zhu, S. Wu, W. Li, CD-HIT: Accelerated for clustering the next-generation sequencing data. *Bioinformatics* **28**, 3150–3152 (2012).

77. F. Chen, K. Wang, J. Kan, M. T. Suzuki, K. E. Wommack, Diverse and unique picocyanobacteria in Chesapeake Bay, revealed by 16S-23S rRNA internal transcribed spacer sequences. *Appl. Environ. Microbiol.* **72**, 2239–2243 (2006).

78. P. D. Schloss, S. L. Westcott, T. Ryabin, J. R. Hall, M. Hartmann, E. B. Hollister, R. A. Lesniewski, B. B. Oakley, D. H. Parks, C. J. Robinson, J. W. Sahl, B. Stres, G. G. Thallinger, D. J. Van Horn, C. F. Weber, Introducing mothur: Open-source, platform-independent, community-

supported software for describing and comparing microbial communities. *Appl. Environ. Microbiol.* **75**, 7537–7541 (2009).

79. Y. Wang, J. Xu, F. Chen, Y. Sun, L. Liu, J. Chen, X. Wang, Y. Hu, B. He, Y. Li, Q. Zheng, Deep learning reveals hidden diversity of *Synechococcus* in the coastal water of China: Novel clades and their ecological insights. *Proc. R. Soc. B Biol. Sci.* **292**, 20251936 (2025).

80. G. V. Uritskiy, J. DiRuggiero, J. Taylor, MetaWRAP—A flexible pipeline for genome-resolved metagenomic data analysis. *Microbiome* **6**, 158 (2018).

81. S. Andrews, FastQC: A quality control tool for high throughput sequence data (Babraham Bioinformatics, 2010); www.bioinformatics.babraham.ac.uk/projects/fastqc/.

82. E. Kopylova, L. Noé, H. Touzet, SortMeRNA: Fast and accurate filtering of ribosomal RNAs in metatranscriptomic data. *Bioinformatics* **28**, 3211–3217 (2012).

83. B. Langmead, S. L. Salzberg, Fast gapped-read alignment with bowtie 2. *Nat. Methods* **9**, 357–359 (2012).

84. S. T. N. Aroney, R. J. P. Newell, J. N. Nissen, A. P. Camargo, G. W. Tyson, B. J. Woodcroft, CoverM: Read alignment statistics for metagenomics. *Bioinformatics* **41**, btaf147 (2025).

85. G. P. Wagner, K. Kin, V. J. Lynch, Measurement of mRNA abundance using RNA-seq data: RPKM measure is inconsistent among samples. *Theory Biosci.* **131**, 281–285 (2012).

86. C. Vernette, J. Lecubin, P. Sánchez, Tara Oceans Coordinators, S. Sunagawa, T. O. Delmont, S. G. Acinas, E. Pelletier, P. Hingamp, M. Lescot, The ocean gene atlas v2.0: Online exploration of the biogeography and phylogeny of plankton genes. *Nucleic Acids Res.* **50**, W516–W526 (2022).

87. A. Torcello-Requena, A. R. J. Murphy, I. D. E. A. Lidbury, F. D. Pitt, R. Stark, A. D. Millard, R. J. Puxty, Y. Chen, D. J. Scanlan, A distinct, high-affinity, alkaline phosphatase facilitates occupation of P-depleted environments by marine picocyanobacteria. *Proc. Natl. Acad. Sci.* **121**, e2312892121 (2024).

88. L. Chen, J. Ma, Y. Huang, M. Dai, X. Li, Optimization of a colorimetric method to determine trace urea in seawater. *Limnol. Oceanogr. Methods* **13**, 303–311 (2015).

89. Y. Shen, R. Benner, A. E. Murray, C. Gimpel, B. Greg Mitchell, E. L. Weiss, C. Reiss, Bioavailable dissolved organic matter and biological hot spots during austral winter in Antarctic waters. *J. Geophys. Res. Oceans* **122**, 508–520 (2017).

90. N. H. Borch, D. L. Kirchman, Concentration and composition of dissolved combined neutral sugars (polysaccharides) in seawater determined by HPLC-PAD. *Mar. Chem.* **57**, 85–95 (1997).

91. R. Benner, Molecular indicators of the bioavailability of dissolved organic matter, in *Aquatic Ecosystems: Interactivity of Dissolved Organic Matter*, S. E. G. Findlay, R. L. Sinsabaugh, Eds. (Academic Press, 2003), pp. 121–137.

92. L. Jørgensen, O. J. Lechtenfeld, R. Benner, M. Middelboe, C. A. Stedmon, Production and transformation of dissolved neutral sugars and amino acids by bacteria in seawater. *Biogeosciences* **11**, 5349–5363 (2014).

93. A. P. Rees, I. Joint, K. M. Donald, Early spring bloom phytoplankton-nutrient dynamics at the Celtic Sea shelf edge. *Deep Sea Res. Part Oceanogr. Res. Pap.* **46**, 483–510 (1999).

94. E. Sahlsten, Nitrogenous nutrition in the euphotic zone of the central north Pacific gyre. *Mar. Biol.* **96**, 433–439 (1987).

95. T. Seemann, Prokka: Rapid prokaryotic genome annotation. *Bioinformatics* **30**, 2068–2069 (2014).

96. L.-T. Nguyen, H. A. Schmidt, A. Von Haeseler, B. Q. Minh, IQ-TREE: A fast and effective stochastic algorithm for estimating maximum-likelihood phylogenies. *Mol. Biol. Evol.* **32**, 268–274 (2015).

97. S. Kalyaanamoorthy, B. Q. Minh, T. K. F. Wong, A. von Haeseler, L. S. Jermiin, ModelFinder: Fast model selection for accurate phylogenetic estimates. *Nat. Methods* **14**, 587–589 (2017).

98. J. Xie, Y. Chen, G. Cai, R. Cai, Z. Hu, H. Wang, Tree visualization by one table (tvBOT): A web application for visualizing, modifying and annotating phylogenetic trees. *Nucleic Acids Res.* **51**, W587–W592 (2023).

99. M. Kanehisa, Y. Sato, M. Kawashima, M. Furumichi, M. Tanabe, KEGG as a reference resource for gene and protein annotation. *Nucleic Acids Res.* **44**, D457–D462 (2016).

100. R. D. Finn, J. Clements, S. R. Eddy, HMMER web server: Interactive sequence similarity searching. *Nucleic Acids Res.* **39**, W29–W37 (2011).

101. G. Gómez-Baena, A. López-Lozano, J. Gil-Martínez, J. M. Lucena, J. Diez, P. Candau, J. M. García-Fernández, Glucose uptake and its effect on gene expression in *Prochlorococcus*. *PLOS ONE* **3**, e3416 (2008).
